# Unveiling Functional‐Metabolic Synergy in the Healthy Brain: Multivariate Integration of Dynamic [ ^18^F]FDG‐PET and Resting‐State fMRI

**DOI:** 10.1002/hbm.70651

**Published:** 2026-09-30

**Authors:** Claudia Tarricone, Giulia Vallini, Giorgia Baron, Massimiliano Facca, Erica Silvestri, Tommaso Volpi, Andrei G. Vlassenko, Manu S. Goyal, Alessandra Bertoldo

**Affiliations:** ^1^ Department of Information Engineering University of Padova Padova Italy; ^2^ Padova Neuroscience Center University of Padova Padova Italy; ^3^ Defitech Chair of Clinical Neuroengineering Neuro‐X Institute (INX), École Polytechnique Fédérale de Lausanne (EPFL) Geneva Switzerland; ^4^ Defitech Chair of Clinical Neuroengineering INX, EPFL Valais Sion Switzerland; ^5^ Department of Radiology and Biomedical Imaging Yale University New Haven Connecticut USA; ^6^ Neuroimaging Laboratories at the Mallinckrodt Institute of Radiology Washington University School of Medicine St Louis Missouri USA

**Keywords:** BOLD frequency bands, dynamic [^18^F]FDG‐PET, functional‐metabolic integration, hemodynamics, partial least squares correlation

## Abstract

Despite accounting for only 2% of body weight, the human brain requires significant amounts of glucose, even at rest, underscoring the importance of functional‐metabolic relationships. Previous studies revealed moderate associations between resting‐state fMRI functional connectivity (FC) and local metabolism via [^18^F]FDG‐PET, yet much remains to be understood, particularly regarding their coupling between functional and metabolic networks. To this end, we employed multivariate Partial Least Squares Correlation (PLSC) to investigate the functional‐metabolic relationship at both nodal and network levels. From dynamic [^18^F]FDG‐PET data we estimated parameters describing glucose metabolism—delivery rate (*K*_1_), phosphorylation rate (*k*_3_), and fractional uptake (*K*_i_)—and generated within‐individual metabolic connectivity (MC) networks. FC was derived from fMRI data filtered into two frequency bands and summarized as region‐wise strength to capture nodal characteristics. Our findings revealed that glucose delivery is linked with FC strength, particularly when fMRI signal frequencies include greater hemodynamic contributions. Even stronger functional‐metabolic coupling occurs at the network level in the low‐frequency fMRI band, with higher MC between sensory/attention and transmodal networks supporting stronger FC within sensory/attention areas. By leveraging PLSC, this work deepens our understanding of the functional‐metabolic synergy in the healthy brain, providing new insights into its organization.

## Key Points

- We identified robust functional‐metabolic synergy in the healthy brain using multivariate Partial Least Squares Correlation (PLSC), revealing strong associations between fMRI‐derived functional connectivity and glucose metabolism from dynamic [^18^F]FDG‐PET.
- At the nodal level, glucose delivery (*K*_1_) is more strongly linked to FC strength than phosphorylation (*k*_3_) or uptake (*K*_i_), especially in fMRI bands enriched with hemodynamic components.
- At the network level, coupling between metabolic and functional connectivity is strongest in the canonical low‐frequency fMRI band, suggesting that integrated metabolic support underpins large‐scale functional integration across brain systems.

## 1 Introduction

The brain's remarkable energy demands, consuming approximately 20% of the body's glucose budget, underscore the critical role of glucose metabolism in sustaining neural function, especially at rest (M. E. Raichle 2006). Understanding the intricate relationship between glucose consumption and functional brain activity is essential for elucidating both normal brain physiology and its dysregulation in disease (Riedl et al. 2016; Park and Friston 2013). Recent advances in neuroimaging have enabled the integration of two powerful techniques: [^18^F]‐fluorodeoxyglucose positron emission tomography ([^18^F]FDG‐PET), which quantifies glucose metabolism (Phelps 2025), and blood oxygenation level‐dependent functional magnetic resonance imaging (BOLD‐fMRI), that provides an indirect measure of neuronal activity through hemodynamic changes (Logothetis 2008; Buxton 2013). BOLD‐fMRI has played a crucial role in investigating local brain activity and functional connectivity (FC) at the network level (Sporns et al. 2005; Behrens and Sporns 2012; Shah et al. 2022; Fox and Raichle 2007). However, neuronal activation is supported by complex neurovascular and neurometabolic processes that are not fully captured by BOLD or FDG measures alone (Raichle and Mintun 2006; Shulman et al. 2009; Maandag et al. 2007; Attwell and Iadecola 2002). Specifically, while BOLD reflects transient fluctuations in oxygen consumption and blood flow driven by synaptic activity (Logothetis et al. 2001), [^18^F]FDG‐PET indexes the metabolic cost of sustaining that activity through glucose utilization (Sokoloff 2008; Vaishnavi et al. 2010). Investigating how the temporal dynamics of neural and hemodynamic processes encoded in the BOLD signal relate to regional glucose metabolism therefore provides a direct window into the neurovascular–metabolic coupling underlying brain function (Riedl et al. 2016; Werner et al. 2015; Mangia et al. 2009). Understanding this coupling can reveal novel insights into brain physiology by linking the energetic demands of neural communication to their vascular and metabolic substrates, potentially elucidating how efficiency, integration, and network dynamics are constrained by energy availability and metabolic support (Saberi et al. 2024; Chen and Zhang 2021; Remme et al. 2018).

Previous studies have examined the relationship between glucose consumption, as measured by [^18^F]FDG‐PET, and various fMRI‐derived features at both local and global levels (Tomasi et al. 2013; Aiello et al. 2015; Palombit et al. 2022; Li et al. 2024; Volpi et al. 2025). Notably, the latest work of Volpi et al. expanded on previous research by analyzing not only semi‐quantitative measures of glucose metabolism, such as the standardized uptake value ratio (*SUVR*), but also the kinetic parameters derived from Sokoloff's model (Volpi et al. 2025). However, while these studies have considered network‐level descriptions in terms of FC or graph‐derived metrics, they lack a network‐level perspective for [^18^F]FDG data. Connectomics has recently shifted its focus towards the molecular processes underlying neural communication, as revealed by PET, with ‘molecular connectivity’ studies (Sala et al. 2023). This approach explores the relationships between PET measures across different brain regions (for [^18^F]FDG: metabolic connectivity (MC)). Over the last few years, some studies have begun to investigate MC in relation to FC. However, most resting‐state investigations have been limited to simple correlation or Dice metrics, which cannot capture the nuanced coupling between metabolic and functional networks (Jamadar et al. 2021; Voigt et al. 2023; Hansen et al. 2023; Volpi et al. 2023). More advanced analytical frameworks have been introduced in task‐based or constant‐infusion fPET studies (Hahn et al. 2024; Hahn et al. 2018), but since they focus on evoked rather than intrinsic activity, they are not directly comparable to the present resting‐state context. Conversely, the recent work by Deery et al. employed a resting‐state fPET protocol with constant infusion and demonstrated that metabolic connectivity exhibits greater predictive utility for age and cognition than functional connectivity, highlighting its sensitivity to age‐related differences and cognitive performance across multiple domains (Deery et al. 2025). This evidence supports the relevance of investigating the coupling between functional and metabolic networks at rest, suggesting that analyses extending beyond simple spatial overlap can yield deeper insights into the neuroenergetic basis of brain network organization. Additionally, previous studies have considered fMRI signals either with high‐pass filtering only (retaining all frequencies above ~0.008 Hz) (Palombit et al. 2022; Volpi et al. 2025; Jamadar et al. 2021) or with band‐pass filtering within the 0.1 Hz bands (Li et al. 2024; Voigt et al. 2023; Castrillon et al. 2023; Shiyam Sundar et al. 2020; Wang et al. 2021), without accounting for the potential impact of different frequency bands, which may carry distinct biological interpretations and levels of information (Yuen et al. 2019; Tong et al. 2019), on their coupling with metabolic counterparts. The different frequency components of the BOLD signal have been linked to distinct neural and physiological processes. Lower frequencies (0.008–0.1 Hz) primarily reflect large‐scale neuronal synchronization and neurovascular fluctuations. In contrast, higher frequencies (up to ~0.2 Hz) encompass faster hamodynamic and vascular oscillations related to autoregulation and metabolic dynamics (Yuen et al. 2019; Tong et al. 2019). Therefore, examining the coupling between metabolism and BOLD fluctuations across multiple frequency ranges may help to distinguish neural contributions from vascular ones, and identify the temporal scales of brain activity that are most closely linked to glucose utilization.

To fill these gaps, we propose a novel approach using Partial Least Squares Correlation (PLSC), a multivariate method that enables the investigation of function‐metabolic coupling at the individual level. Building upon the work by Volpi et al. (2025), who demonstrated that kinetic parameters derived from Sokoloff's model capture complementary aspects of glucose metabolism, *K*_1_ reflecting tracer inflow and vascular delivery, *k*_3_ representing intracellular phosphorylation, and *K*i indexing the net metabolic uptake, we extended this framework to explore how each component relates to functional connectivity. This approach allows the dissociation of vascular, metabolic, and global energetic contributions to functional–metabolic coupling, providing a more physiologically grounded characterization of neuroenergetic organization at rest.

We therefore examined both the nodal coupling between FC strength and [^18^F]FDG kinetic parameters (*K*_1_, *k*_3_, *K*_i_) and the network‐level coupling between FC and MC.

Additionally, we applied the same analyses across distinct frequency bands of the fMRI signal. These include the canonical low‐frequency band (0.008–0.11 Hz), used for BOLD signals to capture low‐frequency neural fluctuations (Thomas Yeo et al. 2011; Kalcher et al. 2012; Mantini et al. 2007; Margulies et al. 2010), and a broader band (0.008–0.21 Hz), which incorporates hemodynamic components (Tong et al. 2019; Chen and Glover 2015). While the first frequency band has previously been shown to have a stronger association with metabolic counterparts (Mantini et al. 2007; Mann et al. 2021), no study to date has directly investigated functional‐metabolic coupling in the second broader band which may better account for potential hemodynamic coupling with [^18^F]FDG metabolism (Tong et al. 2019; Chen and Glover 2015).

This analysis allowed us to explore the interplay between neural activity, hemodynamic, and metabolism, offering a more comprehensive understanding of functional‐metabolic coupling, at both nodal (*K*_1_, *k*_3_, *K*_i_ vs. FC strength) and network (FC vs. MC) levels. In addition, we found that multivariate analysis techniques not only revealed robust relationships but also examined patterns of variability across different brain regions and networks, disentangling their different contributions to the covariation between brain function and metabolism.

## 2 Materials and Methods

### 2.1 Participants

Forty‐two healthy adults (mean age 58.2 ± 14.5 years, 23 females/19 males) underwent [^18^F]FDG‐PET and MRI scans as part of the Adult Metabolism & Brain Resilience (AMBR) study (Goyal et al. 2023). The study adhered to the principles outlined in the Declaration of Helsinki. Approval for all assessments and imaging procedures was obtained from the Human Research Protection Office and the Radioactive Drug Research Committee at Washington University in St. Louis. Written consent was obtained from each participant. Exclusion criteria for participants included contraindications to MRI, history of mental illness, potential pregnancy, or medication use that could affect brain function.

### 2.2 Data Acquisition

High‐resolution structural MRI images were captured using a Siemens Magnetom Prisma^fit^ scanner, employing a 3D sagittal T1‐weighted magnetization‐prepared 180° radio‐frequency pulses and rapid gradient‐echo (MPRAGE) multi‐echo sequence (TE = 1.81, 3.60, 5.39, 7.18 ms, TR = 2500 ms, TI = 1000 ms, voxel size = 0.8 × 0.8 × 0.8 mm^3^). The final T1w image was obtained by averaging the first two echoes (Elam et al. 2021). Additionally, on the same scanner, T2*w gradient‐echo echo planar imaging (GE‐EPI) data (TR/TE = 800/33 ms, flip angle = 52°, voxel size = 2.4 × 2.4 × 2.4 mm^3^, MBAccFactor = 6, a total of 375 volumes acquired over 5 min) and two spin‐echo (SE) acquisitions (TR/TE = 6000/60 ms, FA = 90°, with opposite phase encoding directions (AP, PA)) were acquired.

[^18^F]FDG‐PET imaging was conducted using a Siemens model 962 ECAT EXACT HR+ PET scanner (Siemens/CTI), following intravenous bolus injection of 5.1 ± 0.3 mCi (187.7 ± 12.1 MBq). PET data were dynamically acquired over a duration of 60 min in the eyes‐closed waking state. Participant head movements were minimized using a thermoplastic mask. PET data were reconstructed using filtered back‐projection (ramp filter, 5 mm FWHM) into 128 × 128 × 63 matrices with a voxel size of 2.0 × 2.0 × 2.0 mm^3^. The participant's transmission scan was used to perform the attenuation correction. The reconstruction grid comprised 52 frames of increasing duration, including 24 × 5 s, 9 × 20 s, 10 × 1 min, and 9 × 5 min frames.

### 2.3 Structural MRI Preprocessing

Structural T1w images were corrected for N4 bias field (Tustison et al. 2010), skull‐stripped (Tustison et al. 2013), and segmented into grey matter (GM), white matter (WM), and cerebrospinal fluid (CSF) probability maps using Statistical Parametric Mapping tool (SPM12, https://www.fil.ion.ucl.ac.uk/spm/software/spm12). Subsequently, the T1w images were aligned to the MNI152 FSL standard space via nonlinear diffeomorphic registration using the Advanced Normalization Tools (ANTs, v2.4.3) (Avants et al. 2011).

The present study employed the cortical parcellation of 74 regions of interest (ROI), clustered from the 100‐area Yan homotopic functional atlas (Yan et al. 2023), following the procedure presented in Baron et al. Baron et al. (2025) (https://github.com/FairUnipd/ConsensusClustering‐YanAtlas). This parcellation is chosen because it can effectively capture the symmetric brain uptake of glucose (Shivamurthy et al. 2015) Each ROI was assigned to one of seven Yeo resting‐state networks (RSNs) separately for the left and right hemispheres, resulting in 7 networks per hemisphere (14 networks in total): (Thomas Yeo et al. 2011): Visual network (VIS) (5 parcels), Somatomotor network (SOM) (13 parcels), Dorsal attention network (DAN) (9 parcels), Salience/Ventral attention network (SALVENT) (10 parcels), Limbic network (Limbic) (5 parcels), Control network (CONT) (13 parcels), and Default mode network (DMN/Default) (19 parcels). Additionally, 12 subcortical and cerebellar regions (AAL2 segmentation (Rolls et al. 2015)) were included: six regions per hemisphere, consisting of thalamus, caudate, putamen, pallidum, cerebellum and hippocampus.

### 2.4 Functional MRI Preprocessing

Functional MRI data preprocessing has been described in previous studies (Volpi et al. 2025; Volpi et al. 2023) and follows an approach similar to the Human Connectome Project minimal preprocessing pipeline (Glasser et al. 2013). The first four fMRI volumes were discarded to mitigate non‐equilibrium magnetization effects. Subsequently, the remaining volumes were corrected for slice timing differences (Smith et al. 2004) and magnetic field distortion (Andersson et al. 2003), then realigned to the median volume using FSL's *mcflirt* (Jenkinson et al. 2012). Next, the FSL's ICA‐AROMA (Pruim et al. 2015) approach was used for the removal of spurious variance associated with scanner artifacts. A template EPI volume was obtained from realigned fMRI data using *antsBuildTemplate* (Avants et al. 2011) and used to estimate an affine transform (*flirt*, FSL) employed to map main T1w tissue segmentations and atlas to the EPI space. To remove spurious sources of variability, motion parameters (rotations, translations), their first‐order derivatives, as well as the first five temporal principal components obtained after principal component analysis of WM and CSF EPI signals (Behzadi et al. 2007) were linearly regressed out from all brain voxels in native EPI space (Ciric et al. 2017).

The temporal traces were high‐pass filtered with a cutoff of 0.008 Hz and further low‐pass filtered, resulting in two distinct frequency bands of the original BOLD signal: (i) the canonical band between 0.008–0.11 Hz (referred to as *F*_1_) and (ii) the band between 0.008 and 0.21 Hz (referred to as *F*_2_).

### 2.5 [ ^18^F]FDG‐PET Data Preprocessing and Kinetic Modeling

The dynamic PET scans were motion‐corrected using FSL's *mcflirt* algorithm (Jenkinson et al. 2002). To minimize partial volume effects (PVEs) in regional analyses, no additional spatial smoothing was applied beyond registration and normalization, in line with recent metabolism–flow coupling studies (Cho et al. 2021; Fan et al. 2020; Henriksen et al. 2021; Ishii et al. 2020; Narciso et al. 2021; Wesolowski et al. 2019; Deng et al. 2022; Hyder et al. 2016). This approach, consistent with previously published works on the same dataset (Volpi et al. 2023), was adopted to preserve spatial specificity and reduce cross‐regional signal contamination.

PVEs were further mitigated through a conservative gray matter (GM) masking procedure, in which only voxels with a GM probability ≥ 0.8 (SPM12 segmentation) were retained. This thresholding strategy, validated in the above studies, effectively limits spill‐in and spill‐out from adjacent tissues while preserving an adequate voxel count per region. The same conservative masking was applied for cortical, subcortical, and cerebellar ROIs, using a GM mask for cortical regions and a non‐CSF mask (threshold ≥ 0.8) for subcortical and cerebellar regions.

At individual level, PET kinetic modeling was performed on dynamic data at the voxel level following Volpi et al. (Volpi et al. 2023) An image‐derived input function (IDIF) was extracted from dynamic PET data using a semi‐automatic pipeline, including (1) internal carotid arteries segmentation; (2) selection of “hot voxels” within the vessel mask; (3) parametric clustering to derive the raw IDIF; (4) IDIF model fitting; (5) Chen's spillover correction (Chen et al. 1998) using three late venous samples; (6) delay correction. More details are reported in (Volpi et al. 2023). Voxel‐wise estimation of Sokoloff's model parameters was conducted with a Variational Bayesian inference framework to obtain the parametric maps of blood‐to‐tissue influx rate constant *K*_1_ [ml/cm^3^/min], efflux rate constant *k*_2_ [min^−1^] and the phosphorylation rate constant *k*_3_ [min^−1^]. Then, the parametric maps of the irreversible tracer uptake, *K*_i_ [ml/cm^3^/min], were obtained from the voxel‐level solution of the following equation:

$$K_{i}=\frac{K_{1}k_{3}}{k_{2}+k_{3}}$$

### 2.6 Within‐Individual Features Extraction

For each of the 86 ROIs, pre‐processed rs‐fMRI time‐series, PET time‐activity curves (TACs), and the corresponding ROI parametric values were extracted by averaging voxels within the individual GM mask (details in the Supporting Information Methods). For each participant, two FC matrices—one for each frequency band (*F*_1_ and *F*_2_)—were obtained by computing Pearson's correlation coefficients between the time series of every pair of ROIs. To derive a regional measure of functional connection intensity, these matrices were first thresholded at the 80th percentile, retaining only the top 20% of the correlations. Subsequently, the strength of each node was calculated by summing the values of its connections along the rows of the matrix. The choice of the 80th percentile was motivated by previous literature, which demonstrates that this threshold provides a robust and biologically meaningful balance between sparsity and information preservation when computing node strength from FC matrices (Van Wijk et al. 2010; Van Den Heuvel et al. 2017). This process yielded two vectors of node strength (*FC*_*STR*_) for each participant—one for each frequency band (*FC*_*STR(F1)*_ and *FC*_*STR(F2)*_). Within‐individual MC matrices were computed using the Euclidean Similarity (ES)‐based method (Volpi et al. 2023; Vallini et al. 2025), as briefly outlined in the Supporting Information Methods.

### 2.7 PLSC Multimodal Integration

We applied PLSC to investigate multivariate correlation patterns between various pairs of functional and metabolic measures (Krishnan et al. 2011). Specifically, for both *F*_1_ and *F*_2_ band‐pass filtered FC, we evaluated two levels of multivariate integration: (i) at *nodal‐level*, individual regional values of metabolic parameters *K*_1_, *k*_3_, *K*_i_ have been related to the corresponding *FC*_*STR*_; (ii) at *network‐level*, the edgewise upper triangular features of the full FC and MC matrices have been selected to provide a whole‐network assessment of the functional‐metabolic coupling. For case (i), the matrices used as input for PLSC were organized as participant‐by‐metabolic‐parameter values on one side and participant‐by‐*FC*_*STR*_ on the other. For case (ii), the input matrices were structured as participant‐by‐connection. These participant‐specific input matrices were then z‐scored column‐wise (across participants) to ensure a comparable variability range among participants and features. Thus, the resulting multivariate combinations (*X*–*Y*) were: *K*_1_‐*FC*_*STR*_, *k*_3_‐*FC*_*STR*_, *K*_i_‐*FC*_*STR*_ for nodal‐level investigation, and FC‐MC for the network‐level. PLSC aims to identify orthogonal latent components by maximizing the covariance between two datasets. Each latent component is associated with two singular vectors, V (i.e., X saliences) and U (i.e., Y saliences), which quantify the contribution of each variable (i.e., ROI or matrix entry) to the functional‐metabolic coupling described by that latent component. In the PLSC framework, the linear combinations of the original data variables are weighted by salience maps, resulting in the latent scores. Detailed PLSC equations (Krishnan et al. 2011) are available in the Supporting Information Methods. Figure 1 shows the main steps of PLSC analysis, as implemented by the MATLAB *myPLS* toolbox (v1.0) used here (Zöller et al. 2019), together with the functional and metabolic features considered for nodal or network‐level analysis. The statistical significance of multivariate correlation patterns was assessed using permutation testing (200 permutations, critical value of *α* = 0.01) (Vallini et al. 2025; McIntosh and Lobaugh 2004; Zöller et al. 2017). The reliability of nonzero salience values was assessed by a bootstrapping approach with 100 random samples, calculating standard scores relative to the bootstrap distributions. The significance of the salience vectors was assessed by checking that their 95% confidence interval excluded zero. Rather, PLSC salience matrices were deemed reliable if their absolute standard score exceeded 3 (Griffa et al. 2022). This threshold was set higher than for salience vectors to account for the larger number of variables (Wang et al. 2023).

**FIGURE 1:**
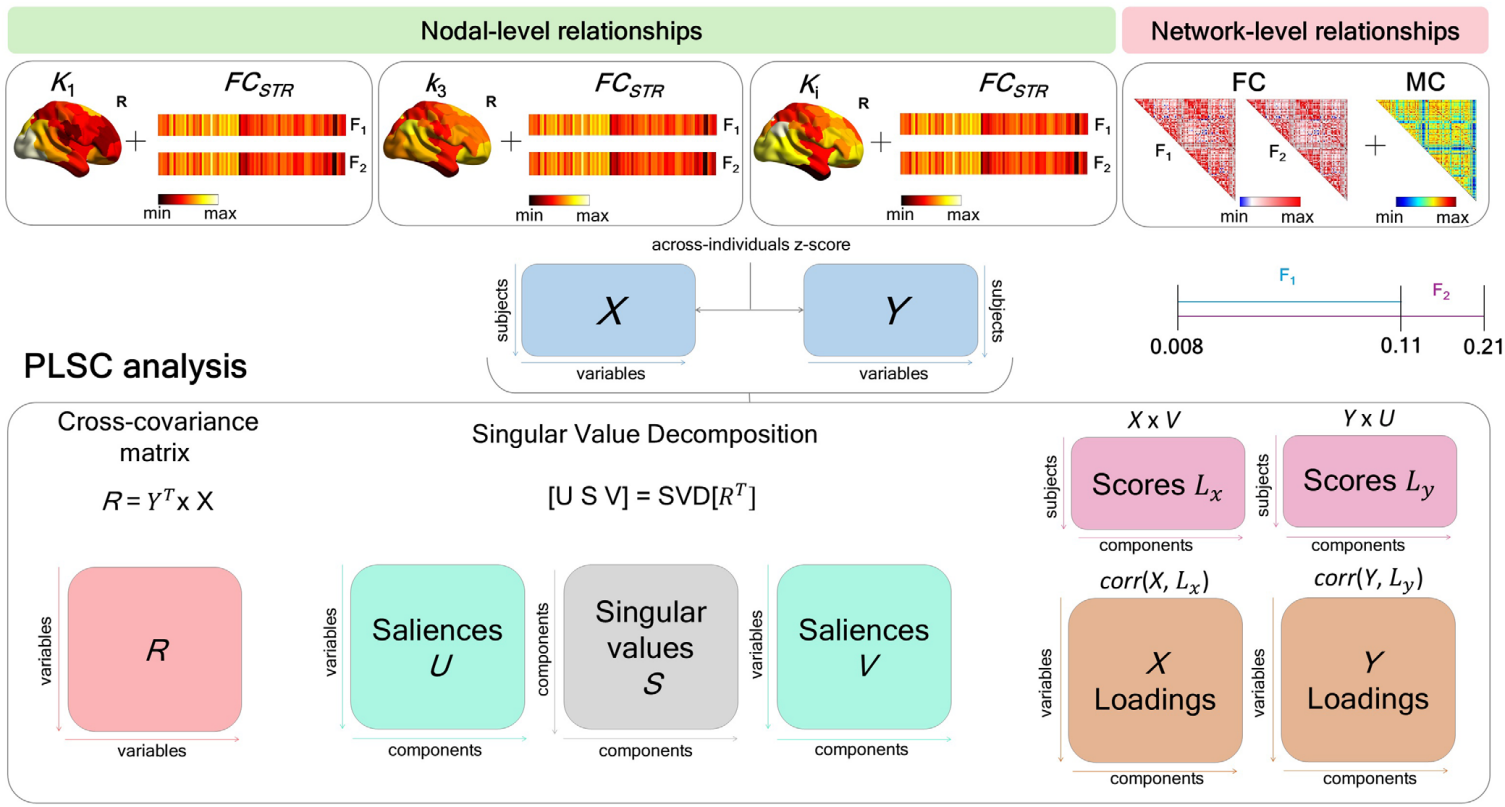
PLSC analysis workflow to assess function‐metabolic coupling. At the nodal level, the relationship was evaluated between *K*_1_, *k*_3_, *K*_i_ and FC node strength (*FC*_*STR*_). At the network level, between the edgewise upper triangles of FC and MC matrices. This workflow was applied twice, considering fMRI features extracted from *F*_1_ (0.008–0.11 Hz) and F_2_ (0.008–0.21 Hz) frequency bands. PLSC computes the cross‐covariance matrix between the original *X* and *Y* data matrices, which is then decomposed using singular value decomposition (SVD) into singular vectors *U* and *V* and singular values *S*. This process identifies latent components (LCs) maximizing the shared covariance between the datasets. The PLSC outputs we evaluated include: (i) saliences, that is the projections of variables along the identified LCs, which describe their relevance to the coupling, and (ii) scores, representing the projection of each participant's variable (*X* or *Y*) onto the respective saliences pattern (*U* or *V*). PLSC also provides *X* and *Y* loadings, computed as Pearson's correlations between the original data (*X* and *Y*) and participant‐specific scores (*L*_*x*_, *L*_*y*_); however, these loadings were not considered in subsequent analyses.

Given the smaller sample size compared to the imaging variables, PLSC models are susceptible to overfitting, resulting in spurious associations that may not generalize well to new data. Therefore, the generalizability of the multivariate correlation patterns obtained from each PLSC analysis was tested using a K‐fold cross‐validation (7 folds, repeated for 100 iterations), retaining 14% of the participants for the test set in each iteration. After excluding the test participants from the dataset, salience maps were estimated using the training set, and the test‐participant data were projected onto these salience maps to obtain the test latent scores (Vallini et al. 2025). The correlation between the original and test latent scores was then evaluated, and the metabolic‐functional pairs were considered generalizable only if the correlation value exceeded 0.5 in at least 50% of the 100 K‐fold cross‐validation repetitions for both *X* and *Y*. Finally, to quantify the relationship between each metabolic‐functional pair in the new latent space, Pearson's correlation between the latent scores (corr(*L*_*x*_,*L*_*y*_)) was computed.

In the Supporting Information Methods, we further show that these results are confirmed and even more significant when increasing the number of permutations and bootstrap resamples to 1000 each. For the main text, however, we report results obtained with 200 permutations and 100 bootstrap samples, which provide a practical balance between reliability and computational efficiency.

### 2.8 PLSC Output Analysis

For generalizable pairs, we conducted a more detailed analysis of the PLSC outputs to assess the contribution of each region/connection to the metabolic‐functional coupling. Since the latent scores reflect the alignment between an individual's original data and the PLSC salience patterns, a positive score indicates a strong similarity between the individual's data and the salience pattern, suggesting a direct relationship. Conversely, a negative score indicates a mismatch or inverse relationship. Thus, from both a metabolic and functional perspective, participants were grouped based on their individual score values, distinguishing between those with positive and negative scores. Statistically significant differences between these two groups were then assessed through statistical tests both at the nodal and network level. For nodal coupling, we compared the value distributions of each brain region with significant salience between the two score groups using a Wilcoxon rank‐sum test (non‐normality assessed via Lilliefors test (Lilliefors 1967)). *p* values were Bonferroni‐corrected. Then, for each ROI, we calculated the median of the parameter values within each group, computed their difference, and masked the results to retain only significantly changed ROIs. For network coupling, we first computed group‐average FC and MC for both score groups, after Fisher z‐transforming the individual matrices (Van Dijk et al. 2010), and we maintain only edges with significant saliences. We then derived difference matrices by subtracting the negative‐score group from the positive‐score group for both FC and MC. A Wilcoxon signed‐rank test (non‐Gaussian distribution confirmed via Lilliefors test (Lilliefors 1967)) was applied to each network‐to‐network relationship, with Bonferroni correction for 36 comparisons. Finally, we generated a block‐difference matrix by computing the median value within each block (excluding non‐significant entries) and retained only those with significant differences. This analysis was performed separately for mean FC (*F*_1_ and *F*_2_) and MC. The choice of different statistical approaches at the nodal and network levels to examine differences between the two groups of participants based on their score values reflects the different data structures. At the nodal level, we aimed to identify specific ROIs with significant group differences. At the network level, given the matrix‐based nature of the data, a difference analysis was more informative for capturing global patterns and network‐level changes, rather than edge‐wise differences. In addition, testing individual edges would have required extensive correction for multiple comparisons, reducing statistical power. Therefore, a network‐based difference approach was statistically more appropriate.

### 2.9 Interpretation of Latent Scores

In the PLSC framework, latent scores represent each participant's position along a continuous latent dimension describing the shared variance between the two datasets. The sign of the latent scores is mathematically arbitrary and depends solely on the orientation of the salience vectors. Accordingly, positive and negative values should not be interpreted as discrete categories but rather as opposite ends of the same latent continuum. In this study, the subdivision of participants into “positive” and “negative” score groups was introduced exclusively as a descriptive tool to visualize and summarize the two opposing patterns of functional–metabolic coupling captured by each latent component. The continuous relationships between the latent scores and the original variables are already represented by the loading values (*corr(X*,*L*_*x*_) and *corr*(*Y*,*L*_*y*_)), which quantify the contribution of each feature to the latent pattern.

## 3 Results

Supplementary Figure 1 shows functional (FC matrices and node strength) and metabolic features (MC matrices and glucose micro/macro‐parameters) averaged across participants.

### 3.1 Differences in the FC Patterns Across Frequency Bands

The connectograph in Figure 2a shows edges where FC differs between the two bands, with red lines indicating stronger FC in *F*_1_ than in *F*_2_ and blue lines representing the opposite pattern. The thickness of the edges is proportional to the mean FC strength of the connected nodes, and the regions are ordered according to their RSN affiliation, with left‐hemisphere nodes appearing before. The distribution of significant differences (|FC_(*F*1)_‐FC_(*F*2)_| > 0.05) reveals an overall FC increase in *F*_1_ compared to *F*_2_ (red edges), particularly within and between networks such as default‐mode network (DMN), limbic, salience/ventral attention (SALVENT), and dorsal attention (DAN) networks. In contrast, blue edges, indicating higher FC in *F*_2_, are fewer and mainly involve connections within the somatomotor (SOM) and visual (VIS) networks. Figure 2b shows the topographic distribution of significant FC strength differences (assessed via Wilcoxon test) between the two frequency bands, with only positive differences (higher *FC*_*STR*_ in F_1_) surviving Bonferroni correction. Increased *FC*_*STR*_ in F_1_ is observed in core DMN regions (posterior cingulate cortex, prefrontal, and temporal areas), the limbic system (orbitofrontal and parahippocampal cortices, temporal pole), and the SALVENT network (insula, medial frontal cortex, parietal operculum). The DAN and SOM networks show significant increases in the postcentral gyrus and motor areas, while subcortical structures (thalamus, caudate, putamen, pallidum, hippocampus) exhibit increased bilateral *FC*_*STR*_. These patterns indicate that frequencies containing more hemodynamic components of the fMRI signal (*F*_2_) preferentially support connectivity in unimodal processing regions (more localized within SOM and VIS domains), while lower frequencies (*F*_1_) enhance large‐scale integration across higher‐order cognitive and associative networks.

**FIGURE 2:**
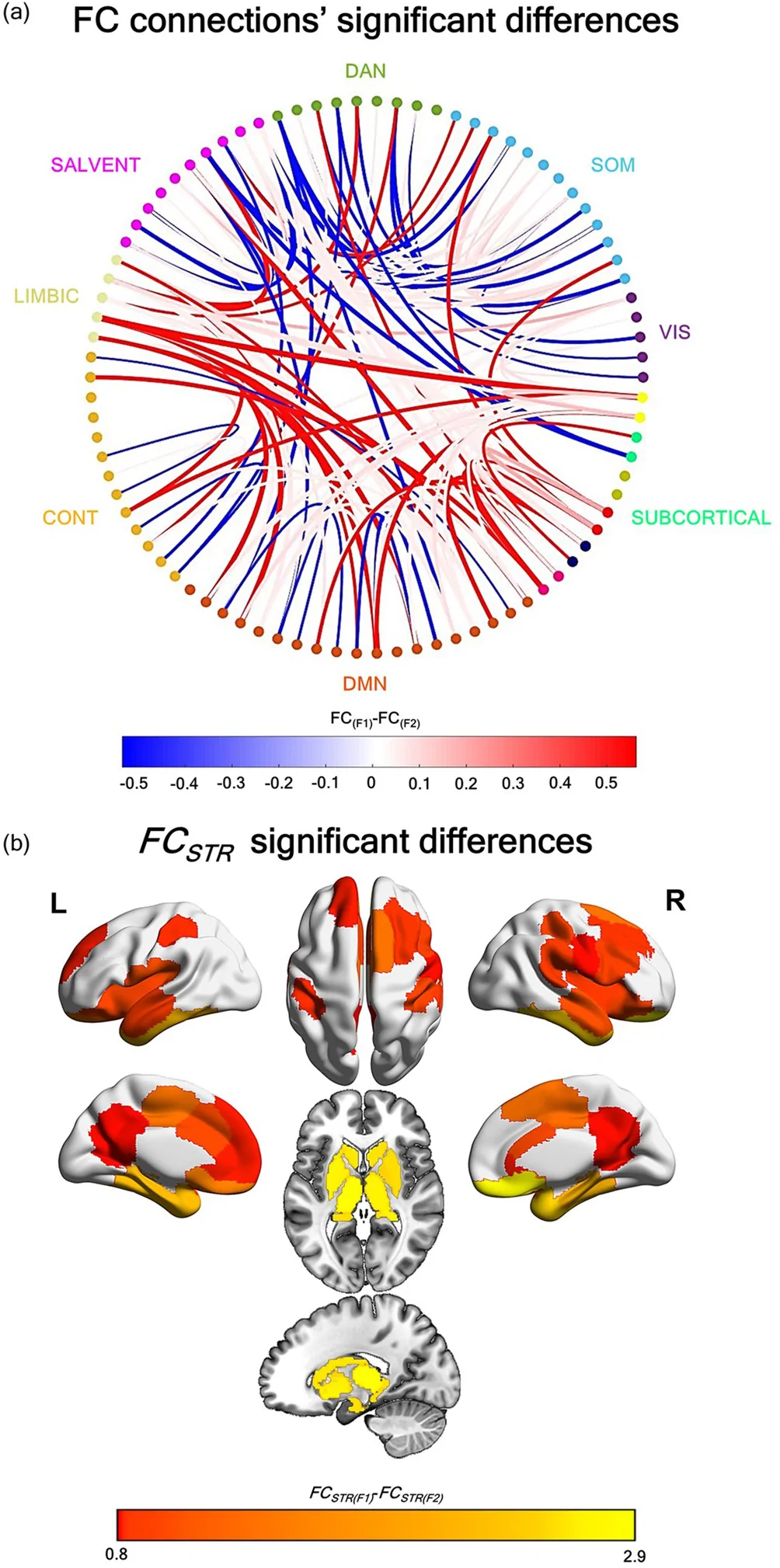
Group‐level differences in FC patterns between the two frequency bands. (a) Circular connectivity graph illustrating the significant differences in FC between frequency bands *F*_1_ and *F*_2_. FC matrices were first Fisher z‐transformed, averaged across participants, and thresholded at the 80th percentile (retaining the top 20% strongest connections). The difference between the thresholded FC matrices was computed, and only connections with an absolute difference greater than 0.05 were retained as significant. Red edges indicate connections where FC in *F*_1_ is greater than in *F*_2_, while blue edges represent connections where FC in *F*_1_ is lower than in *F*_2_. The thickness of each edge is proportional to the average FC strength of the two connected nodes. ROIs are reordered according to their RSNs (within‐network left‐to‐right hemisphere nodes), which are identified by different colors. (b) Topographic maps of the brain regions (within the 86 clustered ROIs atlas) showing significant differences in FC strength between *F*_1_ and *F*_2_, assessed using a Wilcoxon test with Bonferroni correction.

### 3.2 PLSC Multimodal Integration

The results of the generalizability test are reported in Table 1. For generalizable pairs, the table summarizes the average correlation of all values above the threshold, while for non‐generalizable pairs, it includes the average correlation of values below the threshold. Only the *K*_1_‐*FC*_*STR*_ (first latent dimension) and FC‐MC (first and second latent dimensions) pairs were generalizable, while the test failed for *k*_3_‐*FC*_*STR*_ and *K*_i_‐*FC*_*STR*_. Specifically, both nodal and network‐level pairs were generalizable in F_1_ and F_2_ frequency bands, but with different strengths. Indeed, the highest correlation between *X*‐scores and *Y*‐scores (corr(*L*_*x*_,*L*_*y*_)) was found in *F*_2_ for the *K*_1_‐*FC*_*STR*_ pair (*r* = 0.64, *p* < 10^−5^ in *F*_1_; *r* = 0.66, *p* < 10^−5^ in *F*_2_), while for the FC‐MC pair it was observed in F_1_ second latent dimension (*r* = 0.80, *p* < 0^−9^ in *F*_1_; r = 0.78, *p* < 10^−9^ in *F*_2_). Based on the criteria of the highest corr(*L*_*x*_,*L*_*y*_), in subsequent analyses we considered only the PLSC‐identified latent components highlighted in the table. Nodal and network‐level PLSC salience maps are shown in Figure 3.

**TABLE 1:** Results of the generalizability test on the PLSC‐identified relationships.

	Pair	Frequency band	Latent component	corr(*L*_*x*_,*L*_*y*_)	*X* scores generalizability	*Y* scores generalizability
Nodal‐level relationships	*K*_1_‐*FC*_*STR*_	F_1_ (0.008–0.11 Hz)	1	0.641	0.990	0.595
	*k*_3_‐*FC*_*STR*_	F_1_ (0.008–0.11 Hz)	1	0.637	0.997	0.207
	*K*_i_‐*FC*_*STR*_	F_1_ (0.008–0.11 Hz)	1	0.658	0.998	0.047
	*K*_1_‐*FC*_*STR*_	F_2_ (0.008–0.21 Hz)	**1**	**0.657**	**0.989**	**0.607**
	*k*_3_‐*FC*_*STR*_	F_2_ (0.008–0.21 Hz)	1	0.637	0.997	0.369
	*K*_i_‐*FC*_*STR*_	F_2_ (0.008–0.21 Hz)	1	0.590	0.998	0.302

	Pair	Frequency band	Latent component	corr(*L*_*x*_,*L*_*y*_)	*X* scores generalizability	*Y* scores generalizability
Network‐level relationships	FC‐MC	F_1_ (0.008–0.11 Hz)	1	0.745	0.879	0.859
	FC‐MC	F_1_ (0.008–0.11 Hz)	**2**	**0.798**	**0.841**	**0.605**
	FC‐MC	F_2_ (0.008–0.21 Hz)	1	0.738	0.919	0.830
	FC‐MC	F_2_ (0.008–0.21 Hz)	2	0.784	0.698	0.599

*Note:* The upper table reports the results for the nodal‐level analyses, and the lower table contains the results for the network‐level analysis. For each coupling pair, the ordinal number of the significant latent component (LC) derived from the PLSC analysis and the Pearson's correlation value between latent score (corr(*L*_*x*_,*L*_*y*_)) are reported (*p* values < 0.05 in all cases). Values in bold indicate the highest correlation among scores (*p* = 2.25 × 10).

**FIGURE 3:**
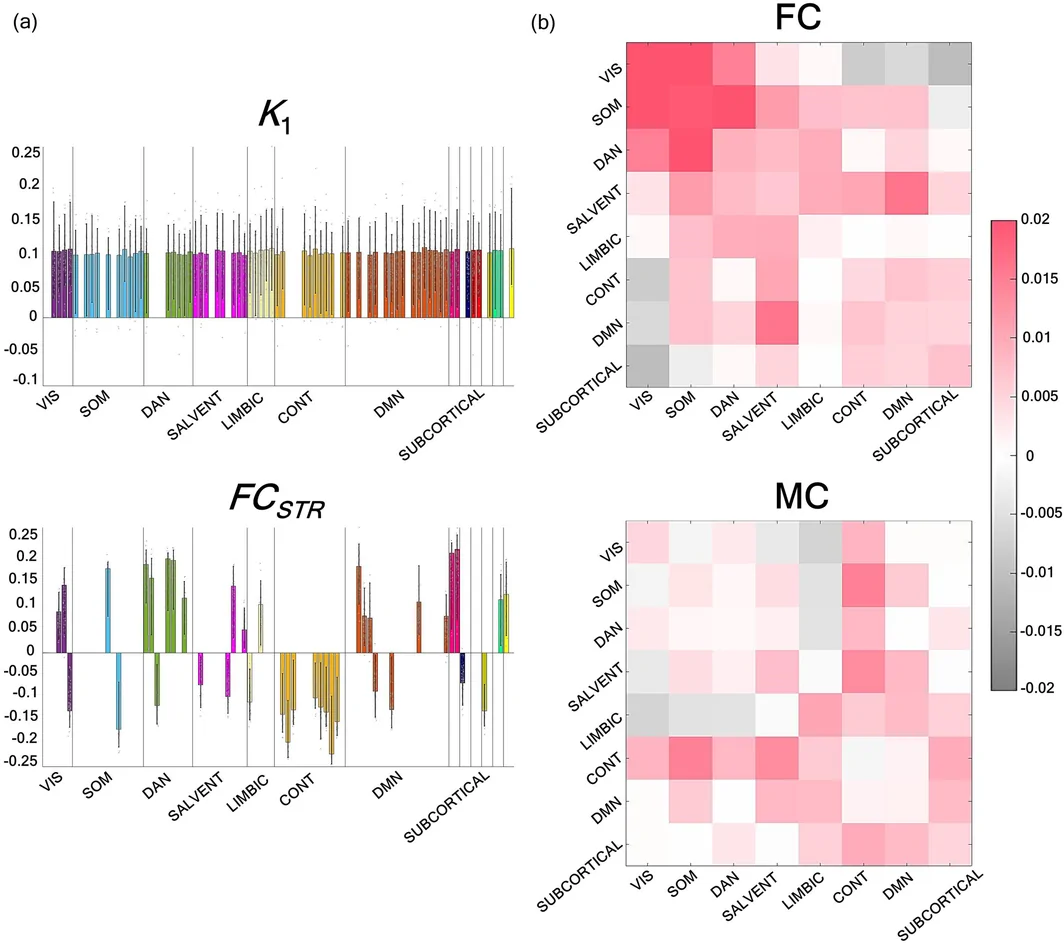
PLSC saliences. (a) Nodal‐level *K*_1_ and *FC*_*STR*_ PLSC saliences, over the bootstrap samples, showing only the salience weights that are significantly different from zero for each network's ROIs. RSNs are identified by different colors. (b) Network level FC‐MC PLSC saliences, reported as the network‐by‐network average of the statistically significant entries values (treating non‐significant saliences as zero, included in the average). While the negative FC saliences between subcortical areas and sensory/attention networks are clearly visible (upper matrix), the same negative entries are less apparent in MC salience pattern (lower matrix) due to the high number of non‐significant entries—assumed to be zero, influencing the average—that characterize this interaction.

### 3.3 Nodal‐Level Functional‐Metabolic Relationships: *K*_1_ ‐ *FC*_*STR*_

At the nodal level (Figure 3a), *K*_1_ saliences (left panel) are uniformly positive and consistent across networks, with relatively homogeneous values. Conversely, *FC*_*STR*_ saliences (right panel) exhibit positive values in some sensory/attention regions (i.e., VIS and DAN network), DMN network, and some subcortical regions (especially the thalamus), while negative values prevail in the control network. Consequently, *K*_1_ and *FC*_*STR*_ saliences align in visual, DAN and DMN networks, while also in some subcortical regions, but diverge in the control network. The salience pattern plays a key role in understanding the coupling characteristics in each individual, as the latent scores reflect the alignment between an individual's original data and the PLSC salience patterns. Therefore, a positive score indicates a strong similarity between the individual's data and the salience pattern. Conversely, a negative score indicates a mismatch or inverse relationship, where the individual's data are more closely aligned with the opposite of the salience pattern.

The scatter plot of *K*_1_‐*FC*_*STR*_ latent scores in Figure 4 illustrates that participants are distributed along a continuous latent axis characterized by two predominant directions of alignment between the *K*_1_ and *FC*_*STR*_ patterns. Since input features were z‐scored across participants, positive and negative latent scores should be interpreted relative to the sample mean, with positive values indicating that an individual's deviations from the mean align with one direction of the latent pattern, and negative values reflecting alignment with the opposite direction. The magnitude of the score reflects the strength of expression of the latent variable, rather than indicating discrete “groups” of individuals. In this context, individuals located in the positive–positive quadrant exhibit metabolic–functional patterns that strongly align with the positive salience maps, while those in the negative–negative quadrant align with the opposite direction of the same latent pattern. For visualization purposes, we divided participants according to the sign of their latent scores to summarize the predominant characteristics of each alignment direction. The resulting masked *K*_1_ and *FC*_*STR*_ maps (*K*_1*(POS)*_, *K*_1*(NEG)*_ *and FC*_*STR(POS)*_, *FC*_*STR(NEG)*_) represent the median values within each alignment direction. Wilcoxon rank‐sum tests indicated that individuals with positive alignment (*K*_1*(POS)*_) displayed significantly higher *K*_1_ values, whereas no significant differences were observed for *FC*_*STR*_ values between the two alignment directions.

**FIGURE 4:**
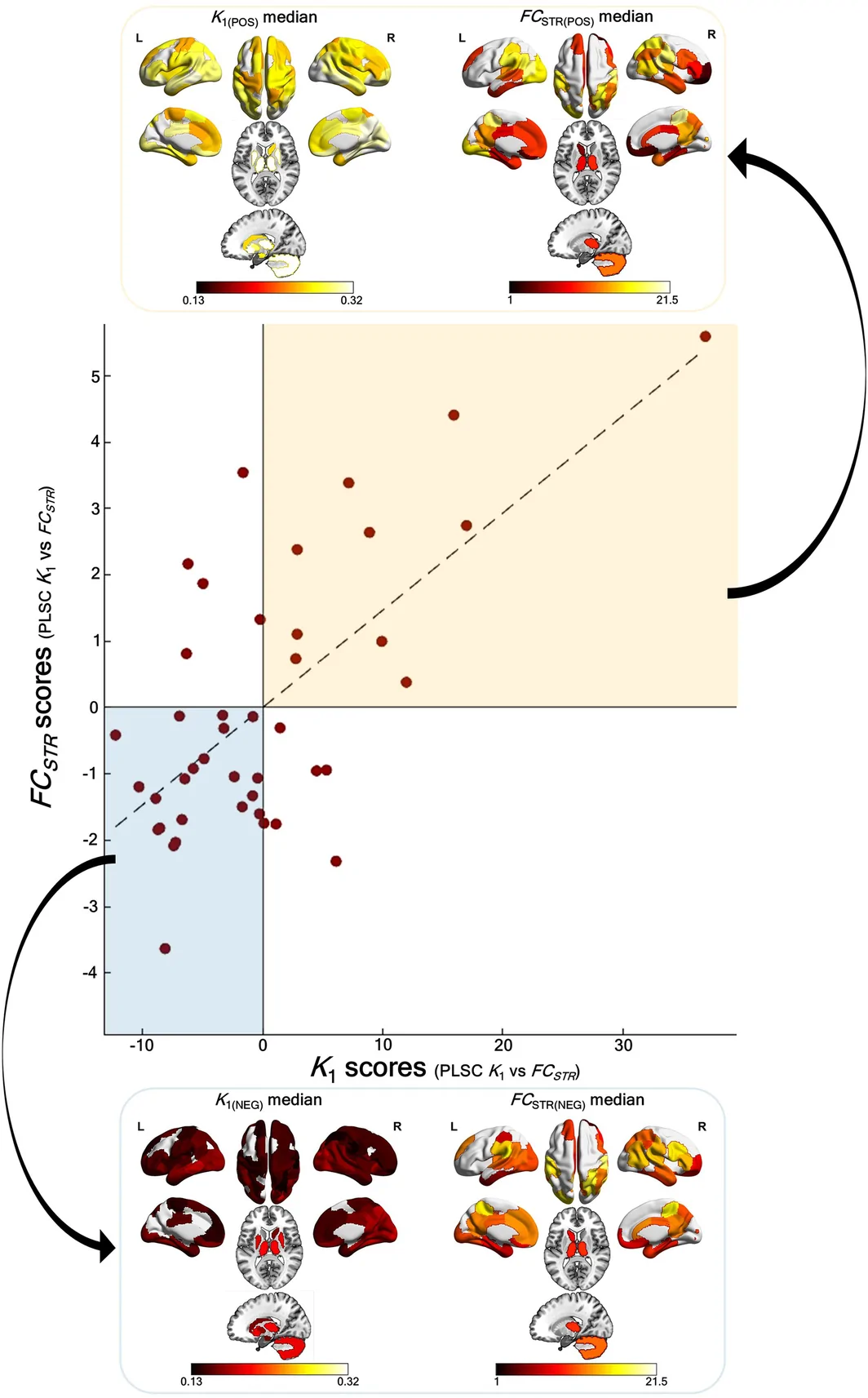
Evaluation of *K*_1_‐*FC*_*STR*_ patterns based on the nodal‐level PLSC scores model. (*Central panel*) Scatter plot of *K*_1_ versus *FC*_*STR*_ PLSC scores, with each dot representing a single participant. The dashed line indicates the least‐square best‐fitting line. The highlighted regions (yellow for positive scores, blue for negative scores) identify the participants who primarily contribute to the variance of the PLSC component, exhibiting distinct metabolic‐functional characteristics. For each group, the median maps of *K*_1_ and *FC*_*STR*_ are visualized, including only the significant values according to the corresponding PLSC salience map. (*Lower panel*) Negative group; (*Upper panel*) Positive group.

### 3.4 Network‐Level Functional‐Metabolic Relationships: FC‐MC

For the FC–MC pair (Figure 3b), we report the network FC and MC salience matrices, that is, the average of entries in each block (including zeros, i.e., non‐significant entries), while full matrices are shown in the Figure S2. The FC salience map reveals that sensory/attention networks (visual, somatomotor, and DAN networks) predominantly exhibit intra‐network positive weights, while transmodal areas display higher weights in inter‐networks connections, especially between salience/ventral attention and DMN networks. Conversely, negative PLSC saliences are more frequent between visual and transmodal networks. In addition, there is a notable reversal in PLSC salience patterns involving subcortical networks, which show predominantly negative weights in the link with sensory/attention networks (except the hippocampus) and positive weights with transmodal networks. The coupling with the metabolic counterpart, as indicated by the MC PLSC‐salience map, is characterized by stronger positive weights between sensory/attention and transmodal networks, while lower positive and more negative weights are observed in intra‐sensory/attention network connections and their associations with the limbic network. Moreover, subcortical areas exhibit a gradient of connectivity, ranging from negative weights with sensory/attention networks (more clearly visible in the full entries matrix provided in Figure S3) to positive weights with transmodal networks, supporting findings from the FC PLSC‐salience map. In the latent space (Figure 5), participants are distributed along a continuous axis with two predominant directions of alignment between FC and MC latent scores. To visualize the main alignment trends, participants were stratified by the sign of their latent scores, yielding average matrices (FC_POS_, FC_NEG_, MC_POS_, and MC_NEG_) summarizing the dominant features of each direction. The positive alignment direction is characterized by stronger FC integration within sensory/attention networks and globally higher MC integration, except for the visual and subcortical areas, which show more segregated MC. Conversely, the negative alignment direction shows weaker within‐network FC (especially in the visual network) and globally weaker inter‐network FC, accompanied by stronger MC integration within sensory/attention regions. Detailed differences are shown in Figure S4, where only the FC mean matrix of the negative alignment direction displayed negative relationships, such as anticorrelations between salience/ventral attention and DMN networks, and between limbic and sensory/attention networks.

**FIGURE 5:**
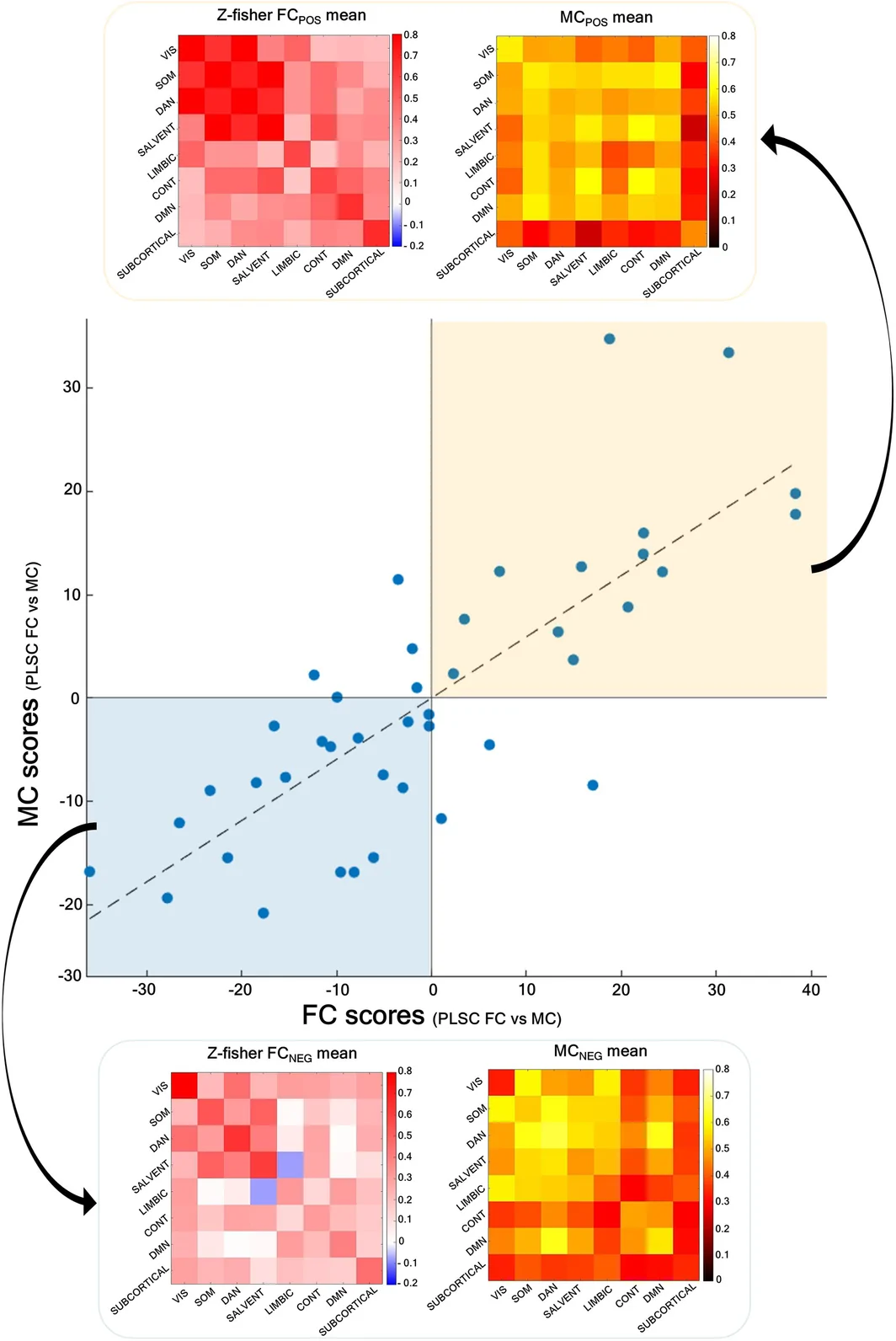
Evaluation of FC‐MC patterns based on the network‐level PLSC scores model. (*Central panel*) Scatter plot of FC versusMC PLSC scores, with each dot representing a single participant. The dashed line indicates the least‐square best‐fitting line. The highlighted regions (yellow for positive scores, blue for negative scores) identify the participants who primarily contribute to the variance of the PLSC component, exhibiting distinct metabolic‐functional characteristics. For each group, the mean FC and MC matrices are visualized, including only the significant FC and MC entries according to the corresponding PLSC salience map, and the network matrices are obtained by averaging (including zeros) the remaining entries in each block (after Fisher z‐transforming the individual matrices). (*Lower panel*) Negative group; (*Upper panel*) Positive group.

Wilcoxon rank‐sum tests (Figure 6) revealed significantly higher FC values for the positive alignment direction, particularly within and between sensory/attention networks and between salience/ventral attention and other networks (except the visual one). These patterns correspond to higher MC values in inter‐network relationships of the control network, especially with somatomotor and salience/ventral attention regions, and to a lesser extent between control, limbic, and DMN networks, while lower MC values were observed between DAN and DMN networks.

**FIGURE 6:**
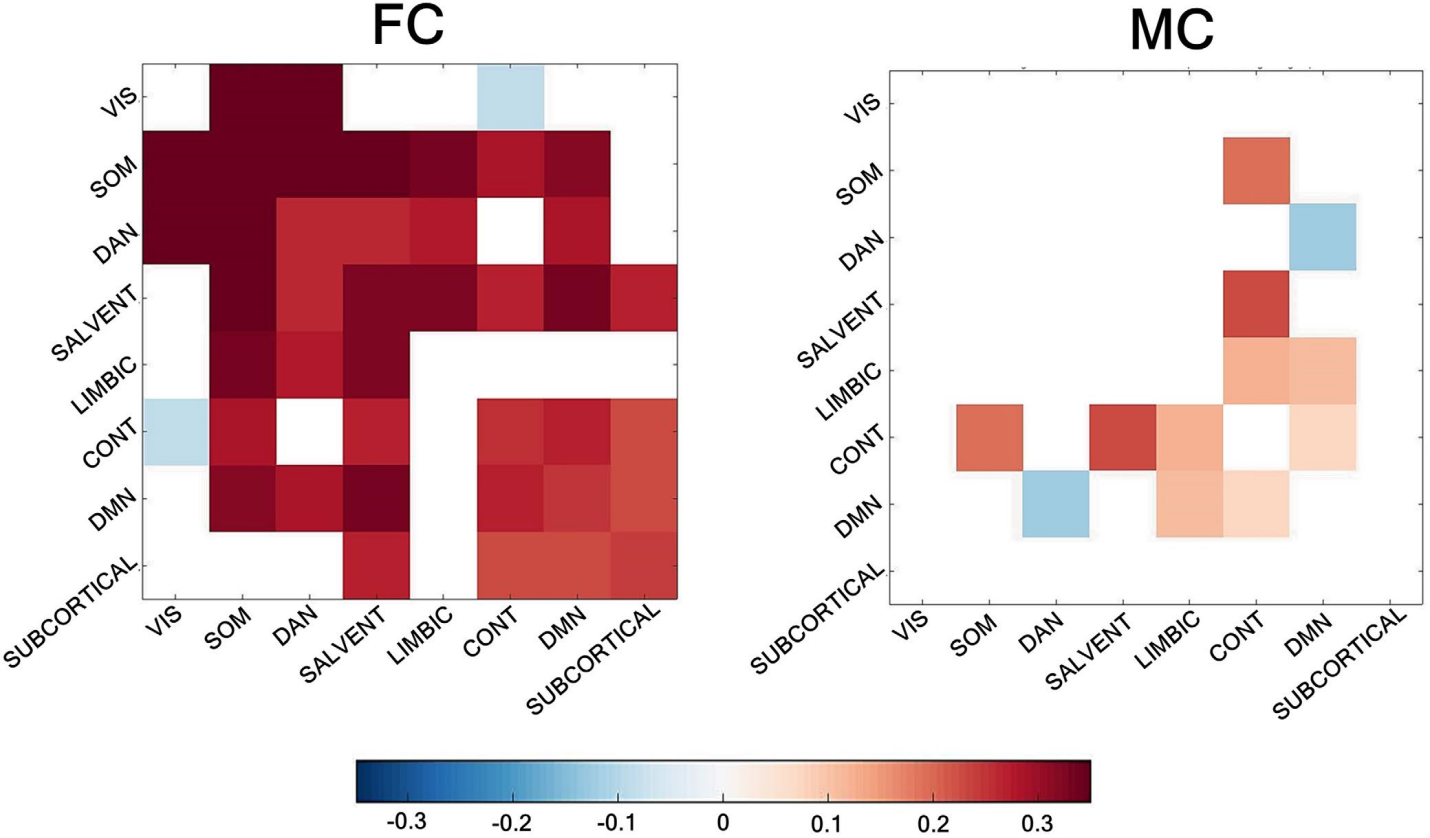
Significant FC and MC inter‐group differences. The figure displays significant differences in functional connectivity (FC, left) and metabolic connectivity (MC, right) computed by subtracting group‐average values (FC_POS_‐ FC_NEG_, MC_POS_‐MC_NEG_), followed by a Wilcoxon signed‐rank test and Bonferroni correction. The block‐difference matrices were generated by calculating the median value of each difference‐matrix block (excluding non‐significant values, as defined by the salience map). Only blocks showing statistically significant differences between the two groups are visualized.

## 4 Discussion

In this study, we investigated the relationship between brain function and metabolism using the multivariate PLSC approach, which is well‐suited for identifying patterns of maximum covariation between two modalities, such as fMRI and PET, while accounting for inter‐individual variability (Volpi et al. 2025). From dynamic PET data, we estimated [^18^F]FDG parameters via kinetic modeling, disentangling the key initial steps of brain glucose metabolism, including delivery (*K*_1_) and phosphorylation by hexokinase (*k*_3_), and the overall fractional irreversible metabolic uptake (*K*_i_), while we also introduced individual‐level metabolic connectivity (MC) matrices generated from dynamic PET TACs (Volpi et al. 2023). Moreover, we investigated the impact of generating FC from fMRI signals filtered into two frequency bands: *F*_1_ (0.008–0.11 Hz) and *F*_2_ (0.008–0.21 Hz). These two frequency bands correspond to distinct temporal scales of spontaneous BOLD signal fluctuations. The *F*_1_ band captures slow oscillations with cycle lengths between approximately 9 and 125 s, which are thought to reflect coherent, large‐scale neural dynamics within canonical resting‐state networks such as the DMN and control systems (Cordes et al. 2001; Zuo et al. 2010). In contrast, the *F*_2_ band extends to faster fluctuations (~5 s per cycle), potentially capturing more transient, localized neural processes (Gohel and Biswal 2015). While these faster components may provide additional information on short‐lived dynamics, they are also more susceptible to physiological noise from cardiac and respiratory sources (Birn et al. 2006; Shmueli et al. 2007).

In the context of functional–metabolic coupling, slower BOLD oscillations are more likely to represent sustained neuronal synchronization that demands higher and more stable glucose delivery, consistent with prior evidence linking low‐frequency BOLD fluctuations with regional glucose metabolism and oxidative processes (Tomasi et al. 2013; Riedl et al. 2014; Stiernman et al. 2023). Conversely, faster BOLD components might reflect transient hemodynamic and neurovascular modulations with weaker metabolic coupling, potentially capturing fluctuations less tightly constrained by energy consumption. Therefore, exploring functional–metabolic associations across these two frequency bands provides insight into how glucose metabolism supports both stable and transient modes of intrinsic brain activity.

We hypothesized that different frequency components of the fMRI signal capture distinct neurophysiological processes, which may differentially relate to metabolic activity. This was particularly relevant for the *F*_2_ band, where the BOLD signal exhibits a greater hemodynamic contribution (Yuen et al. 2019; Tong et al. 2019), potentially affecting its association with [^18^F]FDG kinetic parameters. To explore this, we examined the functional‐metabolic relationship at both the nodal (*K*_1_, *k*_3_, *K*_i_ vs. *FC*_*STR*_) and the network (FC vs. MC) level, across the *F*_1_ and *F*_2_ frequency bands.

### 4.1 Nodal‐Level Functional‐Metabolic Relationships: *K*_1_ ‐ *FC*_*STR*_

A nodal coupling emerged between *K*_1_ and *FC*_*STR*_, suggesting that glucose delivery processes play a more significant role in modulating this relationship with the functional counterpart than either glucose phosphorylation (*k*_3_) or the tracer's irreversible uptake (*K*_i_). This finding is consistent with the results of Volpi et al. Volpi et al. (2025) who showed that *K*_1_ is the only [^18^F]FDG parameter to exhibit significant bivariate associations with fMRI features (graph measures) related to the hemodynamic response function (HRF) and static FC. Their findings, which highlighted non‐linearities and substantial inter‐individual variability in the strength of association between metabolic parameters and static FC features, including FC strength, may also explain the limited generalizability of the *k*_3_‐*FC*_*STR*_ and *K*_i_‐*FC*_*STR*_ relationships. Here, we found that the *K*_1_‐*FC*_*STR*_ relationship is stronger in the *F*_2_ band, which captures higher‐frequency BOLD fluctuations, thereby incorporating more hemodynamic information. Additionally, the *F*_2_ band encompasses the frequency range (0.13–0.17 Hz), most likely associated with low‐frequency vascular oscillations (or vasomotion) (Yuen et al. 2019). According to recent studies (Volpi et al. 2025; Wu and Marinazzo 2016), perfusion and BBB permeability, as reflected by *K*_1_, are crucial contributors to the rs‐fMRI signal and may be more closely associated with large‐scale FC than with glucose metabolism. Having confirmed our hypothesis that the hemodynamic contribution to the fMRI signal might reveal a stronger relationship with metabolic measures reflecting glucose delivery from blood to tissue, we further explored the spatial patterns of these associations using PLSC outputs. This allowed us to examine whether there were differences in the relationship between function and glucose metabolism, both among brain regions belonging to different functional networks (and thus involved in different domains), and between study participants.

Specifically, the joint analysis of saliences and latent scores facilitates the characterization of individual participant profiles, providing more insight into the combined metabolic‐functional nodal coupling. From the examination of the corresponding *K*_1_–*FC*_*STR*_ latent scores (Figure 4), two distinct categories of individuals contributing to the variance of the component can be identified. The first group consists of individuals with positive latent scores, characterized by predominantly elevated *K*_1_ values across the brain, reflecting high glucose uptake. In this group, high *FC*_*STR*_ values are observed in sensory/attention regions, posterior parcels of the DMN, and the thalamus, while lower *FC*_*STR*_ values are seen in prefrontal cortex areas of the control network. Conversely, the second group of individuals (negative latent scores) displays predominantly lower *K*_1_ values, alongside high *FC*_*STR*_ in the control network while lower in the remaining regions.

This pattern likely reflects the role of the DMN, sensory/attention systems, and thalamus in information processing and integration (Tomasi et al. 2013; M. E. Raichle 2015), processes that are known to require increased glucose supply (Xu et al. 2023). Therefore, our data reveal a subject‐level association wherein individuals with high *FC*_*STR*_ in these regions tend to show correspondingly high *K*_1_ values, while those with low *FC*_*STR*_ exhibit low *K*_1_. This suggests a consistent positive coupling between functional connectivity strength and glucose delivery across distinct participant profiles, aligning with previous studies linking stronger functional connectivity to higher resting cerebral blood flow or FDG SUVR in these regions (Tomasi et al. 2013; Riedl et al. 2014; Xu et al. 2023; Liang et al. 2013).

Interesting is the opposite direction between *FC*_*STR*_ and *K*_1_ in the control network. In the subgroup, characterized by high *K*_1_ and low *FC*_*STR*_ values (positive scores group), the pattern may reflect a reduced number of strong functional correlations (i.e., lower strength) between the control network and other large‐scale systems—suggesting a functional architecture that favors localized intra‐network processing over broad‐scale integration. This interpretation is supported by thresholded mean FC matrices (Figure S2), which highlight enhanced intra‐network control connectivity in these individuals. The elevated *K*_1_ values in control regions may indicate a metabolic cost associated with maintaining internal coordination and network readiness, even in the absence of widespread functional integration. Conversely, the subgroup displaying low *K*_1_ and high *FC*_*STR*_ in the control network (negative scores group) may reflect a metabolically efficient configuration optimized for flexible communication across networks. In this case, the control network appears to dynamically integrate information from externally oriented attentional systems (e.g., DAN) and internally driven networks (e.g., DMN) (Menon 2013; Menon and D'Esposito 2022), while requiring relatively lower glucose supply. The reduced *K*_1_ values in control regions may thus reflect lower localized metabolic demands, potentially due to a shift away from sustained intra‐network signaling toward more efficient, distributed functional coordination (Ciric et al. 2017; Bullmore and Sporns 2012). Details on the quantitative assessment of the differences observed between intra‐ and inter‐control network relationships in the FC matrices (Figure S3) are provided in the Supporting Information Discussion. Moreover, by focusing specifically on glucose delivery, as indexed by *K*_1_, our analysis provides more direct insight into the vascular and transport mechanisms that support functional interactions, independently of downstream metabolic utilization.

However, despite the presence of a trend across participants transitioning from one group to another, statistically significant differences are evident only for *K*_1_ values between the two groups, while no significant differences emerge in terms of *FC*_*STR*_. This reflects the limited variability of *FC*_*STR*_ values between the two groups, which measure the strength of regional interactions but do not account for the specific connectivity patterns driving these interactions. These observations emphasize the sensitivity of multivariate approaches like PLSC in capturing fine‐grained coupling patterns that may not be detectable with simpler bivariate tests. However, while a clear coupling between *K*_1_ and *FC*_*STR*_ exists, nodal‐level analysis is inherently constrained by the use of a surrogate connectivity measure (e.g., *FC*_*STR*_).

### 4.2 Network‐Level Functional‐Metabolic Relationships: FC‐MC

Our analyses revealed that the relationship between FC and MC was stronger compared to the one between *FC*_*STR*_ and *K*_1_, reinforcing the initial hypothesis that glucose metabolism and fMRI‐derived FC exhibit robust coupling when examined at the network level rather than through local/nodal metabolic measures alone (Volpi et al. 2025). Additionally, a more robust FC‐MC coupling is observed within the *F*_1_ band, consistent with prior evidence associating glucose metabolism to slow‐frequency fMRI fluctuations within the 0.063–0.098 Hz band (Yuen et al. 2019; M. E. Raichle 2011). Notably, our approach extends recent work by Li et al. (2024), which used canonical correlation analysis (CCA) to link FC and regional SUVR measures. While they reported network‐level associations between functional connectivity and regional metabolism, our MC framework provides a more direct comparison with FC and reveals how distributed metabolic patterns support system‐level functional communications. Specifically, we found that the control network appears to play a pivotal role in this context, acting as a metabolic and functional bridge between sensory/attention and transmodal networks, a finding consistent with its established role in modulating attention and cognitive control, while also maintaining the balance between externally oriented and internally oriented processes (Nejati et al. 2021; Nejati et al. 2023; Harms and Pollak 2024). This expands on Li et al.'s observations by demonstrating that metabolic coupling, not just static local glucose uptake, is integral to large‐scale functional network dynamics.

Stratifying participants by scores' sign (Figure 5) further underscored this relationship: the positive group exhibited highly integrated FC within sensory/attention networks and globally distributed MC (except for visual/subcortical areas, which showed metabolic segregation), whereas the negative group displayed weaker FC (particularly in visual networks) but stronger MC integration within sensory/attention regions. This functional–metabolic association aligns with previous findings suggesting that stable and sustained FC patterns within extrinsic networks, such as those involved in sensory and attentional processing, are typically supported by increased metabolic demands. Our results extend this observation by highlighting that such metabolic support is not merely local but may be mediated by the interaction between sensory/attention and transmodal networks, possibly to sustain the synchronous functional activity of sensory/attentional systems. This interpretation is further supported by Figure 6, which highlights group‐level differences (both in MC and FC) and underscores the coupling between functional activation in sensory/attentional areas and robust metabolic support from the control network, suggesting a key role of the latter as a metabolic orchestrator for attention and large‐scale integration processes.

### 4.3 Limitations

Despite the innovative findings presented in this study, it is important to acknowledge that fMRI and PET acquisitions were not simultaneous, which may limit the ability to capture concurrent metabolic and functional changes. Additionally, hemodynamic responses are influenced by several physiological and external factors, including hydration status (Billington et al. 2018; Watso and Farquhar 2019), caffeine intake (Gaspar et al. 2024; Pelligrino et al. 2010), sleep patterns (Csipo et al. 2021; Hajjar et al. 2007; Qin et al. 2020; Alex et al. 2017), and the time of day (Aguirre et al. 1998; Vaisvilaite et al. 2022) at which the scan is performed. However, the relationship between metabolism and function using such a dataset has been explored in previous studies (Volpi et al. 2025; Goyal et al. 2023; Vallini et al. 2025). The present analyses were performed without partial volume correction (PVC) of the PET data. To assess the robustness of this choice on MC‐based network measures, we conducted a dedicated sensitivity analysis by applying the Iterative Yang PVC method (Erlandsson et al. 2012; Thomas et al. 2016) as implemented in PETPVC (Thomas et al. 2016) and recomputing MC matrices using the same Euclidean Similarity approach. PVC introduced a statistically significant global increase in MC values (+7.26%; t(41) = 6.182, *p* < 0.001) and genuine pattern changes beyond simple intensity rescaling (mean *R* ^2^ = 0.458; mean residual SD = 0.116), with the largest effects anatomically localized to cortico‐subcortical connections involving structures with known high PVE susceptibility, Hippocampus (max relative change: 94%), Pallidum (up to 38%), and Caudate (up to 39%) (Quarantelli et al. 2004). Critically, despite these quantitative differences, the overall rank‐order of pairwise connectivity was substantially preserved across subjects (mean Spearman *r* = 0.639, all *p* < 0.001), and the spatial distribution of PVC‐induced changes is fully consistent with known PVE physiology. Full results are reported in the Supporting Information (Figure S5).

Importantly, PVC could not be applied to the kinetic microparameters *K*_1_ and *k*_3_, nor to the macroparameter *K*_i_, estimated via our Variational Bayesian quantification framework. In this pipeline, tissue‐specific anatomical priors restrict parameter estimation to grey and white matter voxels, excluding extra‐cerebral tissues. The peripheral signal thereby excluded is precisely what is required for accurate PSF modelling in post hoc PVC (Erlandsson et al. 2012), rendering any post hoc correction mathematically biased and incomplete (Reader and Zaidi 2007; Tsoumpas et al. 2008). Since PVC cannot be consistently applied across all quantitative measures derived from the PET data, and given the overall consistency of MC results before and after PVC demonstrated by the sensitivity analysis, we opted not to apply PVC to the MC matrices in the main analysis. Future work integrating PVC prospectively within the kinetic modelling step, rather than as a post hoc procedure, would be valuable for a more complete characterization of PVEs on microparametric measures and their derived network metrics.

Furthermore, this study focused exclusively on healthy individuals and did not incorporate cognitive or clinical measures. Consequently, the predictive value and potential clinical relevance of the proposed coupling metrics must be established through future work integrating behavioral or patient data (Vallini et al. 2025).

## 5 Conclusions

In summary, our results demonstrate the capability of PLSC to uncover covariation patterns between brain function and metabolism at both nodal and network levels, with notable differences based on the BOLD signal band‐pass filtering. Specifically, nodal coupling between *K*_1_ and FC strength is stronger in the frequency band enriched with hemodynamic information. In contrast, network‐level relationships, which were also the strongest among all the tested pairs, are more pronounced in the resting state canonical frequency band, associated with BOLD signal frequencies more reflective of metabolic processes. These findings provide compelling evidence for a robust network‐level coupling between brain function and metabolism, highlighting the limitations of nodal‐level analyses (Palombit et al. 2022; Volpi et al. 2025). Notably, nodal‐level coupling appears to be more influenced by directionality of signal interactions (Vallini et al. 2025), while the network level gains a more nuanced understanding of the variability in functional‐metabolic relationships. Additionally, deepening our understanding of how metabolic energy supports brain functionality has been proven to be essential for unravelling the complexities of brain disorders. By exploring how these intricate relationships are disrupted in pathological conditions, as exemplified in prior research on gliomas using a comparable framework, we can gain critical insights into the specific brain regions responsible for such alterations, ultimately shedding light on the underlying mechanisms that distinguish healthy from diseased states (Vallini et al. 2025).

## Author Contributions

Andrei G. Vlassenko and Manu S. Goyal collected the data. Claudia Tarricone, Giulia Vallini, and Alessandra Bertoldo designed the research. Claudia Tarricone, Giulia Vallini, Giorgia Baron, Massimiliano Facca, Erica Silvestri and Tommaso Volpi analyzed the data. Claudia Tarricone, Giulia Vallini, Giorgia Baron, Massimiano Facca, Erica Silvestri, Tommaso Volpi, Andrei G. Vlassenko, Manu S. Goyal, and Alessandra Bertoldo interpreted the results. Claudia Tarricone and Giulia Vallini wrote the manuscript. All authors revised the manuscript.

## Funding

The research was supported by #NEXTGENERATIONEU (NGEU) and funded by the Ministry of University and Research (MUR), National Recovery and Resilience Plan (NRRP), project MNESYS (PE0000006)—A Multiscale integrated approach to the study of the nervous system in health and disease (DN. 1553 11.10.2022).

## Conflicts of Interest

The authors declare no conflicts of interest.

## Supporting information


**Data S1:** Supporting Informations

**Table S1:** Participants demographic information.

**Figure S1:** Group‐average functional and metabolic features. Dynamic [^18^F]FDG‐PET data were used to estimate (a) FDG micro/macro‐parameters (*K*_1_, *k*_3_ and *K*_i_) at nodal level (86 ROIs) and (c) Metabolic Connectivity (MC) matrices at network level (7 RSNs per hemisphere plus 12 SUBCORTICAL regions). From rs‐fMRI data, both in F_1_ and F_2_ band, we derived (c) Functional Connectivity (FC) matrices at network level (7 RSNs per hemisphere plus 12 SUBCORTICAL regions) and (b) FC strength (*FC*_*STR*_) for each of the 86 ROIs. Brain nodes were grouped based on RSNs (left‐hemisphere nodes (L) first, followed by right‐hemisphere ones (R)). VIS = visual network; SOM = somatomotor network; DAN = Dorsal attention network; SALVENT = Salience/Ventral attention network; LIMBIC = Limbic network; CONT = Control network; DMN = Default mode network; SUBCORTICAL = thalamus, caudate, putamen, pallidum, cerebellum and hippocampus.

**Figure S2:** Full matrices of PLSC saliences at network‐level. The colors of the entries correspond to the magnitude and sign of the associated significant salience value (i.e., red for positive and grey for negative saliences' values), with non‐significant entries in white (according to the bootstrap procedure described in the Methods section).

**Figure S3:** Fisher‐z mean and 80th percentile‐thresholded FC in the F_2_ band. The figure displays the mean 80th percentile‐thresholded FC matrices for the group of *K*_1_‐*FC*_*STR*_ negative scores' (on the left) and for the group of *K*_1_‐*FC*_*STR*_ positive scores' (on the right), obtained by averaging the FC in the F_2_ band of the subjects belonging to each of the scores' groups.

**Figure S4:** Full‐entries scores' groups average FC and MC matrices. (a) Mean FC (on the left) and MC (on the right) matrices for the group of subjects with positive FC‐MC scores (a) and with negative FC‐MC scores (b), obtained including only the significant FC and MC entries according to the corresponding PLSC salience map (non‐significant values are shown in white).

**Figure S5:** Sensitivity analysis: effect of partial volume correction (PVC) on MC matrices (*n* = 42 subjects; *n* = 3655 pairwise connections). (A) Group‐average difference matrix (post−pre PVC; diverging red–blue colormap centered at zero), showing the spatial distribution of PVC‐induced changes across all 86 ROIs. Warm colors indicate connections with increased MC after correction; cool colors indicate decreased MC. (B) Distribution of per‐subject Spearman rank correlations between pre‐ and post‐PVC MC vectors ((red line: group mean *r* = 0.639; dashed line: *r* = 0.5 reference threshold); 4 out of 42 subjects showed *r* < 0.5; all *p* values < 0.0001). (C) Scatter of group‐average pre‐ vs. post‐PVC MC values for all pairwise connections, colored by symmetric relative change (hot colormap); solid line: mean subject‐level regression fit (slope = 0.582, intercept = 0.246); dashed line: identity. The systematic deviation from the identity line reflects both a scale change and an additive offset introduced by PVC. (D) Distribution of mean symmetric relative change per connection (|post−pre|/(0.5 · |post + pre|)); white line: mean (25.3%); dashed line: median. (E) Symmetric matrix of mean relative change per connection; the concentration of high‐change values along rows and columns corresponding to subcortical ROIs is clearly visible. (F) Top 15 ROIs ranked by mean relative change averaged across all their connections; color intensity reflects the magnitude of change. Subcortical structures (Caudate, Pallidum, Hippocampus) consistently show the largest PVC‐induced corrections, consistent with their known high susceptibility to partial volume effects due to small volume and grey matter heterogeneity. Cortical nodes appearing among the most affected ROIs (Cont_pCun, Default_PCC_RSC) reflect their disproportionate share of cortico‐subcortical connections rather than intrinsic cortical PVE.

## Data Availability

The data that support the findings of this study are available from the corresponding author upon reasonable request. The data are not publicly available due to privacy or ethical restrictions.
