# Supplementary material for: Unveiling Functional‐Metabolic Synergy in the Healthy Brain: Multivariate Integration of Dynamic [ 18F]FDG‐PET and Resting‐State fMRI
Source: Hum Brain Mapp. 2026 Sep 30;47(14):e70651. doi: 10.1002/hbm.70651 (PMC13626976; doi:10.1002/hbm.70651)
Supplement: Supplementary file 1 [file HBM-47-e70651-s003.docx]

*Participants demographic information*

| **ID** | **Dose** | **Age** | **Sex** | **Weigh** | **Height** | **BMI** | **BSA** | **Gluc** | **HbA1c** | **EstGl** | **Insulin** |
| --- | --- | --- | --- | --- | --- | --- | --- | --- | --- | --- | --- |
| 108004 | 5,4 | 58 | M | 86,18 | 1,70 | 29,76 | 2,29 | 110 | 5,6 | 114 | n/a |
| 108013 | 5,5 | 65 | M | 108,86 | 1,83 | 32,55 | 2,72 | 95 | 5,1 | 100 | n/a |
| 108016 | 5 | 54 | F | 56,70 | 1,70 | 19,58 | 1,92 | 67 | 4,9 | 94 | n/a |
| 108029 | 4,9 | 45 | F | 86,18 | 1,68 | 30,67 | 2,26 | n/a | n/a | n/a | n/a |
| 108036 | 5,25 | 49 | F | 58,97 | 1,75 | 19,20 | 2,01 | 94 | 4,9 | 94 | n/a |
| 108038 | 5,1 | 68 | F | 99,79 | 1,70 | 34,46 | 2,44 | 87 | 5,1 | 100 | n/a |
| 108040 | 5,3 | 44 | F | 102,06 | 1,75 | 33,23 | 2,53 | 103 | 5,4 | 108 | n/a |
| 108044 | 5,3 | 66 | F | 68,04 | 1,68 | 24,21 | 2,04 | 95 | 5,4 | 108 | 4,8 |
| 108045 | 4,77 | 41 | F | 77,56 | 1,63 | 29,35 | 2,09 | 88 | 5,2 | 103 | 10,3 |
| 108046 | 5,36 | 56 | M | 66,22 | 1,70 | 22,87 | 2,05 | 75 | 5,2 | 103 | 8,5 |
| 108050 | 5 | 35 | F | 72,57 | 1,75 | 23,63 | 2,19 | 86 | 4,7 | 88 | 5,9 |
| 108170 | 5 | 76 | M | 86,18 | 1,73 | 28,89 | 2,32 | 99 | 5,6 | 114 | 10,3 |
| 108174 | 5,3 | 55 | F | 81,65 | 1,63 | 30,90 | 2,14 | 101 | 5,7 | 117 | 12,1 |
| 108179 | 5 | 71 | F | 96,62 | 1,63 | 36,56 | 2,30 | 89 | 5,2 | 103 | 5,3 |
| 108181 | 4,6 | 76 | M | 86,18 | 1,68 | 30,67 | 2,26 | 117 | 5,9 | 123 | 25,5 |
| 108183 | 5,14 | 81 | F | 63,50 | 1,70 | 21,93 | 2,01 | 90 | 4,8 | 91 | 5,1 |
| 108187 | 5,1 | 69 | F | 87,54 | 1,57 | 35,30 | 2,13 | 96 | 6,3 | 134 | 13,6 |
| 108191 | 5 | 71 | M | 76,20 | 1,73 | 25,54 | 2,21 | 93 | 4,9 | 94 | 10,5 |
| 108199 | 5 | 70 | F | 56,70 | 1,52 | 24,41 | 1,72 | 88 | 5,1 | 100 | 8 |
| 108200 | 4,8 | 68 | F | 78,47 | 1,68 | 27,92 | 2,17 | 99 | 5,6 | 114 | 29,2 |
| 108202 | 5 | 68 | F | 75,30 | 1,65 | 27,62 | 2,10 | 87 | 5,1 | 100 | 6,1 |
| 108205 | 5 | 65 | F | 50,80 | 1,57 | 20,48 | 1,69 | 91 | 4,9 | 94 | 8,2 |
| 108208 | 5,2 | 74 | F | 67,13 | 1,60 | 26,22 | 1,94 | 177 | 7,2 | 160 | 38,2 |
| 108214 | 5,2 | 63 | F | 65,77 | 1,63 | 24,89 | 1,95 | 83 | 5,1 | 100 | 4,4 |
| 108222 | 5,3 | 68 | M | 112,94 | 1,75 | 36,77 | 2,65 | 98 | 4,9 | 94 | 16,1 |
| 108227 | 4,8 | 25 | F | 70,31 | 1,88 | 19,90 | 2,32 | 87 | 5,2 | 103 | 5 |
| 108228 | 4,8 | 57 | F | 58,97 | 1,70 | 20,36 | 1,95 | 108 | 5,5 | 111 | 7,6 |
| 108229 | 4,02 | 27 | F | 74,84 | 1,75 | 24,37 | 2,22 | 88 | 4,7 | 88 | 5,8 |
| 108230 | 5,2 | 76 | F | 77,11 | 1,73 | 25,85 | 2,22 | 101 | 5,7 | 117 | 15,2 |
| 108231 | 5,2 | 25 | M | 86,18 | 1,55 | 35,90 | 2,08 | 95 | 4,4 | 80 | 25,4 |
| 108237 | 5,2 | 62 | M | 92,08 | 1,70 | 31,79 | 2,36 | 95 | 5,2 | 103 | 12,6 |
| 108238 | 5,2 | 71 | F | 72,57 | 1,60 | 28,34 | 2,00 | 89 | 5,2 | 103 | 6,8 |
| 108241 | 5,1 | 62 | M | 77,11 | 1,70 | 26,63 | 2,18 | 95 | 5,2 | 103 | 7,5 |
| 108244 | 5,2 | 56 | M | 72,57 | 1,68 | 25,82 | 2,10 | 101 | 5,1 | 100 | 3,3 |
| 108251 | 5,7 | 61 | F | 86,18 | 1,78 | 27,26 | 2,39 | 98 | 5,5 | 111 | 4,8 |
| 108262 | 5,1 | 65 | M | 113,40 | 1,78 | 35,87 | 2,69 | 117 | 6,1 | 128 | 24,5 |
| 108281 | 5,3 | 49 | M | 113,40 | 1,83 | 33,91 | 2,77 | 102 | 5,6 | 114 | 11,8 |
| 108283 | 4,8 | 39 | M | 86,18 | 1,78 | 27,26 | 2,39 | 101 | 5 | 97 | 7,1 |
| 108289 | 5,3 | 45 | M | 90,72 | 1,80 | 27,89 | 2,48 | 105 | 5,6 | 114 | 21 |
| 108291 | 4,9 | 55 | M | 70,31 | 1,75 | 22,89 | 2,16 | 99 | 5,6 | 114 | 7,1 |
| 108293 | 5,1 | 42 | M | 88,45 | 1,88 | 25,04 | 2,56 | 72 | 5,1 | 100 | 13,4 |
| 108295 | 5,3 | 51 | M | 81,65 | 1,78 | 25,83 | 2,34 | 90 | 5,9 | 123 | 6,6 |
