# Supplementary material for: Unveiling Functional‐Metabolic Synergy in the Healthy Brain: Multivariate Integration of Dynamic [ 18F]FDG‐PET and Resting‐State fMRI
Source: Hum Brain Mapp. 2026 Sep 30;47(14):e70651. doi: 10.1002/hbm.70651 (PMC13626976; doi:10.1002/hbm.70651)
Supplement: Supplementary file 2 [file HBM-47-e70651-s006.docx]

**Supplementary Materials for “Unveiling functional-metabolic synergy in the healthy brain:**

**multivariate integration of dynamic [^18^F]FDG-PET and resting-state fMRI”**

Claudia Tarricone, Giulia Vallini, Giorgia Baron, Erica Silvestri, Tommaso Volpi,

Andrei G. Vlassenko, Manu S. Goyal, Alessandra Bertoldo*

*Correspondence should be addressed to

Alessandra Bertoldo, Ph.D.

Via Gradenigo 6/B

35122 Padova, Italy

[alessandra.bertoldo@unipd.it](mailto:alessandra.bertoldo@unipd.it)

**Supplementary Materials**

*Participants demographic information*

| **ID** | **Dose** | **Age** | **Sex** | **Weigh** | **Height** | **BMI** | **BSA** | **Gluc** | **HbA1c** | **EstGl** | **Insulin** |
| --- | --- | --- | --- | --- | --- | --- | --- | --- | --- | --- | --- |
| 108004 | 5,4 | 58 | M | 86,18 | 1,70 | 29,76 | 2,29 | 110 | 5,6 | 114 | n/a |
| 108013 | 5,5 | 65 | M | 108,86 | 1,83 | 32,55 | 2,72 | 95 | 5,1 | 100 | n/a |
| 108016 | 5 | 54 | F | 56,70 | 1,70 | 19,58 | 1,92 | 67 | 4,9 | 94 | n/a |
| 108029 | 4,9 | 45 | F | 86,18 | 1,68 | 30,67 | 2,26 | n/a | n/a | n/a | n/a |
| 108036 | 5,25 | 49 | F | 58,97 | 1,75 | 19,20 | 2,01 | 94 | 4,9 | 94 | n/a |
| 108038 | 5,1 | 68 | F | 99,79 | 1,70 | 34,46 | 2,44 | 87 | 5,1 | 100 | n/a |
| 108040 | 5,3 | 44 | F | 102,06 | 1,75 | 33,23 | 2,53 | 103 | 5,4 | 108 | n/a |
| 108044 | 5,3 | 66 | F | 68,04 | 1,68 | 24,21 | 2,04 | 95 | 5,4 | 108 | 4,8 |
| 108045 | 4,77 | 41 | F | 77,56 | 1,63 | 29,35 | 2,09 | 88 | 5,2 | 103 | 10,3 |
| 108046 | 5,36 | 56 | M | 66,22 | 1,70 | 22,87 | 2,05 | 75 | 5,2 | 103 | 8,5 |
| 108050 | 5 | 35 | F | 72,57 | 1,75 | 23,63 | 2,19 | 86 | 4,7 | 88 | 5,9 |
| 108170 | 5 | 76 | M | 86,18 | 1,73 | 28,89 | 2,32 | 99 | 5,6 | 114 | 10,3 |
| 108174 | 5,3 | 55 | F | 81,65 | 1,63 | 30,90 | 2,14 | 101 | 5,7 | 117 | 12,1 |
| 108179 | 5 | 71 | F | 96,62 | 1,63 | 36,56 | 2,30 | 89 | 5,2 | 103 | 5,3 |
| 108181 | 4,6 | 76 | M | 86,18 | 1,68 | 30,67 | 2,26 | 117 | 5,9 | 123 | 25,5 |
| 108183 | 5,14 | 81 | F | 63,50 | 1,70 | 21,93 | 2,01 | 90 | 4,8 | 91 | 5,1 |
| 108187 | 5,1 | 69 | F | 87,54 | 1,57 | 35,30 | 2,13 | 96 | 6,3 | 134 | 13,6 |
| 108191 | 5 | 71 | M | 76,20 | 1,73 | 25,54 | 2,21 | 93 | 4,9 | 94 | 10,5 |
| 108199 | 5 | 70 | F | 56,70 | 1,52 | 24,41 | 1,72 | 88 | 5,1 | 100 | 8 |
| 108200 | 4,8 | 68 | F | 78,47 | 1,68 | 27,92 | 2,17 | 99 | 5,6 | 114 | 29,2 |
| 108202 | 5 | 68 | F | 75,30 | 1,65 | 27,62 | 2,10 | 87 | 5,1 | 100 | 6,1 |
| 108205 | 5 | 65 | F | 50,80 | 1,57 | 20,48 | 1,69 | 91 | 4,9 | 94 | 8,2 |
| 108208 | 5,2 | 74 | F | 67,13 | 1,60 | 26,22 | 1,94 | 177 | 7,2 | 160 | 38,2 |
| 108214 | 5,2 | 63 | F | 65,77 | 1,63 | 24,89 | 1,95 | 83 | 5,1 | 100 | 4,4 |
| 108222 | 5,3 | 68 | M | 112,94 | 1,75 | 36,77 | 2,65 | 98 | 4,9 | 94 | 16,1 |
| 108227 | 4,8 | 25 | F | 70,31 | 1,88 | 19,90 | 2,32 | 87 | 5,2 | 103 | 5 |
| 108228 | 4,8 | 57 | F | 58,97 | 1,70 | 20,36 | 1,95 | 108 | 5,5 | 111 | 7,6 |
| 108229 | 4,02 | 27 | F | 74,84 | 1,75 | 24,37 | 2,22 | 88 | 4,7 | 88 | 5,8 |
| 108230 | 5,2 | 76 | F | 77,11 | 1,73 | 25,85 | 2,22 | 101 | 5,7 | 117 | 15,2 |
| 108231 | 5,2 | 25 | M | 86,18 | 1,55 | 35,90 | 2,08 | 95 | 4,4 | 80 | 25,4 |
| 108237 | 5,2 | 62 | M | 92,08 | 1,70 | 31,79 | 2,36 | 95 | 5,2 | 103 | 12,6 |
| 108238 | 5,2 | 71 | F | 72,57 | 1,60 | 28,34 | 2,00 | 89 | 5,2 | 103 | 6,8 |
| 108241 | 5,1 | 62 | M | 77,11 | 1,70 | 26,63 | 2,18 | 95 | 5,2 | 103 | 7,5 |
| 108244 | 5,2 | 56 | M | 72,57 | 1,68 | 25,82 | 2,10 | 101 | 5,1 | 100 | 3,3 |
| 108251 | 5,7 | 61 | F | 86,18 | 1,78 | 27,26 | 2,39 | 98 | 5,5 | 111 | 4,8 |
| 108262 | 5,1 | 65 | M | 113,40 | 1,78 | 35,87 | 2,69 | 117 | 6,1 | 128 | 24,5 |
| 108281 | 5,3 | 49 | M | 113,40 | 1,83 | 33,91 | 2,77 | 102 | 5,6 | 114 | 11,8 |
| 108283 | 4,8 | 39 | M | 86,18 | 1,78 | 27,26 | 2,39 | 101 | 5 | 97 | 7,1 |
| 108289 | 5,3 | 45 | M | 90,72 | 1,80 | 27,89 | 2,48 | 105 | 5,6 | 114 | 21 |
| 108291 | 4,9 | 55 | M | 70,31 | 1,75 | 22,89 | 2,16 | 99 | 5,6 | 114 | 7,1 |
| 108293 | 5,1 | 42 | M | 88,45 | 1,88 | 25,04 | 2,56 | 72 | 5,1 | 100 | 13,4 |
| 108295 | 5,3 | 51 | M | 81,65 | 1,78 | 25,83 | 2,34 | 90 | 5,9 | 123 | 6,6 |

Supplementary Table S.1: Participants’ demographic and cardiometabolic health information. Dose [MBq/kg]: Injected dose of [¹⁸F]FDG normalized to body weight. Age [years]: Participant’s chronological age at the time of the scan. Sex [M/F]: Biological sex of the participant. Weight [kg]: Body weight measured before the scan. Height [cm]: Body height of the participant. BMI [kg/m²]: Body mass index, calculated as weight divided by height squared. BSA [m²]: Body surface area, estimated from height and weight (DuBois) formula. Gluc [mmol/L]: Fasting blood glucose concentration measured prior to tracer injection. HbA1c [%]: Glycated hemoglobin, indicating average blood glucose over the previous months. EstGl [mmol/L]: Estimated average glucose derived from HbA1c values. Insulin [µU/mL]: Fasting plasma insulin concentration.

**Supplementary Methods**

*Functional MRI preprocessing*

Each subject was assigned a binary temporal mask to identify brain volumes corrupted by head motion (Framewise Displacement > 0.4 mm). This mask was utilized to adjust the reliability of temporal frames, censoring the data with high-motion frames and checking that each subject had a minimal number of 200 remaining volumes after censoring^1^.

*Common fMRI and PET preprocessing steps*

Preprocessed rs-fMRI signals, along with the regional parametric maps and TACs from dynamic PET images were extracted for each of the total 86 defined ROIs by averaging the voxel activities within the GM SPM mask (a threshold of 0.8 was applied to the probability maps to generate binary masks) for cortical voxels and the non-cerebrospinal fluid (notCSF) SPM mask (threshold of 0.8) for subcortical and cerebellum voxels. The selected segmentation approach was intentionally conservative, yielding a mean sample time course that effectively mitigates partial volume effects (PVEs) while still retaining an adequate number of voxels.

*Within-individual features extraction*

For each pair of TACs, z_1_ and z_2_ , their Euclidean distance is calculated as:

|  |  |  |
| --- | --- | --- |

where *T* is the number of time points. An index of the ES is derived by subtracting 1 from the normalized Euclidean distance between each regional TAC pair, which is then divided by the maximum distance between TAC pairs, thereby scaling it within the interval [0, 1]. Because of the skewed distribution of ES values, a Fisher z-transformation was applied, followed by a subsequent rescaling of values within the range [0, 1]. This metric has been proven to reveal a network structure in wi-MC matrices, as expected from brain connectivity studies^2^.

*Partial Least Square Correlation (PLSC) equations*

As first, the relationship between the two input matrices is quantified by calculating the dot product of the columns of X and Y, given by:

$$R=Y^{T}X$$

resulting in the cross-product matrix that represents the correlation between the z-scored columns of the X and Y matrices, indicating the correlation between brain functional and metabolic measures. If the columns of the matrices are only centred but not normalized, the *R* matrix reflects covariance. The cross-product matrix, R, undergoes singular value decomposition (SVD[R^T^])^3,4^, resulting in:

$$U * S * V^{T}=R$$

The decomposition of R^T^ yields a set of mutually orthogonal latent components (LCs), with U and V being orthogonal matrices containing left and right singular vectors, respectively, and S being a diagonal matrix of singular values. The number L of latent variables equals the rank of the covariance matrix R, which is the smallest of its dimensions. Each latent variable is associated with: (1) a singular value (on the diagonal of S) indicating how much of the cross-block covariance (R) is explained by that latent variable; (2) a vector of singular values V, representing the X saliences; (3) a vector of singular values U, representing the Y saliences. The first and subsequent LCs account for the greatest and progressively decreasing amounts of the cross-covariance matrix (indicated by the square of the singular value), respectively. The saliences indicate how strongly each variable (ROI or matrix entry) contributes to the functional-metabolic correlation explained by a given latent variable. Projecting (using the dot product) each subject’s original data (in X or Y) onto the multivariate salience pattern (in V or U, respectively) yields a set of functional-metabolic latent scores $L_{X}=X * V$ and $L_{Y}=Y * U$, measuring the similarity of each individual’s brain data with the corresponding salient pattern. Finally, functional and metabolic structure coefficients (or “loadings”) can be computed as Pearson’s correlations between the original data (X and Y) and subject-specific scores (*L_x_*, *L_y_*). Loadings indicate the extent of contribution of each variable to the latent variable derived from the PLS analysis. In summary, PLS utilizes latent variables to capture the most significant shared information between two different modalities of brain data by maximizing covariance. **Figure 2.** shows the main steps of PLSC analysis, as implemented by the MATLAB *myPLS* toolbox (v1.0) used in this study^5^.

*Additional stability analysis with increased permutations and bootstrap samples*

To assess the robustness of the PLSC results with respect to the number of resampling iterations, we repeated the analysis for the functional–metabolic pairs that were found to be generalizable in cross-validation. Specifically, the PLSC linking *K*_1_ and FC strength (F_2_ band) was rerun using 1000 permutations and 1000 bootstrap resamples (compared to the 200/100 settings used in the main analysis). The same latent component (LC1) remained significant and exhibited a lower empirical p-value (0.001 vs. 0.005), indicating increased stability. The permutation-derived null distribution also became smoother and more densely sampled with 1000 permutations, confirming that the original result was not driven by insufficient resampling but reflects a robust underlying multivariate pattern. Similarly, for the network-level functional–metabolic pair (FC–MC), all analyses were repeated with 1000 permutations and 1000 bootstrap resamples. In this case, LC2 remained the most significant component, with its empirical p-value decreasing from 0.035 to 0.002, further confirming the stability and robustness of the observed latent components at the network level.

*The effect of PVC on MC matrices: a sensitivity analysis*

To assess the robustness of MC-based network findings to partial volume effects, we applied the Iterative Yang PVC method^6,7^ as implemented in PETPVC^8^ to all individual PET images, and recomputed the MC matrix for each subject using the same Euclidean Similarity approach as in the main analysis.

To evaluate (i) whether PVC shifts MC intensity globally and (ii) whether the connectivity pattern is preserved after correction, we conducted a sensitivity analysis comprising two complementary approaches. First, we computed the Spearman rank correlation between pre- and post-PVC upper-triangular MC vectors at the subject level, to quantify the degree of rank-order preservation in pairwise connectivity values. Second, we performed a per-subject linear regression of post-PVC on pre-PVC MC values (post = a + b · pre), estimating the slope b, intercept a, and coefficient of determination R², to characterise the nature of the correction (multiplicative, additive, or mixed). We additionally computed the mean symmetric relative change per connection, defined as |post − pre| / (0.5 · |post + pre|), and identified the ROIs and connections showing the highest rate of change following PVC correction. Results are summarised in **Supplementary Figure S.5**.

**Supplementary Results**

*The effect of PVC on MC matrices: a sensitivity analysis*

PVC introduced a statistically significant global increase in MC values of +7.26% (mean pre-PVC: 0.498 ± 0.026; mean post-PVC: 0.534 ± 0.032; paired t-test: t(41) = 6.182, p < 0.001 **Supplementary Figure S.5A**). A global intensity increase following PVC is consistent with the well-established phenomenon of signal recovery in regions affected by partial volume averaging, where uncorrected PET values systematically underestimate true tracer concentration due to the limited spatial resolution of the scanner^9–11^. Because MC values in this dataset are strictly positive, the signed and absolute analyses yield identical results, confirming that the shift is systematic and unidirectional.

At the subject level, the mean Spearman correlation between pre- and post-PVC MC patterns was r = 0.639 ± 0.124 (median = 0.669; range: 0.179–0.805), with only 4 out of 42 subjects showing r < 0.5, and all individual correlations statistically significant (all p < 0.001; **Supplementary Figure S.5B**). Per-subject regression of post-PVC on pre-PVC MC (post = a + b · pre) yielded a mean slope b = 0.582 ± 0.124 (significantly different from 1: p < 0.001), a mean intercept a = 0.246 ± 0.058 (significantly different from 0: p < 0.001), and a mean R² = 0.458 ± 0.132 (mean residual SD = 0.116 ± 0.013; **Supplementary Figure S.5C**). This is consistent with previous reports showing that PVC affects brain regions non-uniformly depending on their size, shape, and local grey matter fraction^6,7,12^. These results indicate that PVC introduces genuine pattern changes beyond simple intensity rescaling: approximately 54% of the variance in post-PVC MC values is not linearly explained by pre-PVC MC. The mean symmetric relative change per connection was 25.3% ± 9.7% (**Supplementary Figure S.5D**).

The largest connection-wise changes were concentrated in cortico-subcortical connections: the most affected connection (RH_Cont_pCun – RH_Hippocampus) showed a relative change of 94%, followed by Putamen–Hippocampus connections (up to 88%), and connections linking posterior cingulate and precuneus regions to hippocampal and pallidal structures (range: 69–87%; Fig. S.5E). At the ROI level, the structures driving the largest corrections were the Caudate (LH: 39.0%, RH: 35.8%), Pallidum (LH: 37.4%, RH: 38.4%), and Hippocampus (LH: 35.0%, RH: 36.9%), structures with well-documented high PVE susceptibility due to their small size and grey matter heterogeneity ^13–15^ (**Supplementary Figure S.5F**). Among the ten most affected ROIs, four were cortical nodes: RH_Cont_pCun (35.3%), LH_Cont_pCun (33.2%), RH_Default_PCC_RSC (33.7%), and LH_Default_PCC_RSC (32.3%), consistent with their role as hubs of cortico-subcortical connectivity^16,17^ rather than reflecting intrinsic cortical PVE.

Taken together, these results confirm that while PVC quantitatively alters MC values, with the largest effects anatomically localised to structures known for high PVE susceptibility, the overall rank-order of pairwise connectivity is substantially preserved across the majority of subjects (mean Spearman r = 0.639; **Supplementary Figure S.5B**), and the spatial distribution of PVC-induced changes is fully consistent with known PVE physiology^6^.

**Supplementary Discussion**

Specifically, to provide a meaningful and quantitative assessment of the differences observed between intra- and inter-control network relationships in the matrices shown in **Supplementary Figure S.3**, the distributions of intra- and inter-control FC values were compared via Wilcoxon rank sum test, both within the same score group (intra vs. inter -FC within the positive or negative group) and between the two score groups. Notably, and in line with the results of **Supplementary Figure S.3**, significant differences emerged when comparing intra-control FC values between the two groups (p-value = 0.012) and when comparing intra- and inter-control FC within the positive score group (p-value = 10^-4^). This supports our idea that lower *FC_STR_* values in control regions compared to the average are primarily due to lower inter-network connections of these regions, which are compensated by higher intra-network FC values. Conversely, no significant differences were observed between intra- and inter-control FC in the negative score group (p-value = 0.089), nor when comparing inter-control FC values between the two groups (p-value = 0.534). This suggests that the higher *FC_STR_* values in the negative scores group, relative to the average, are due to a balance between intra- and inter-network connectivity, both of which contribute to the overall higher *FC_STR_*.

**Supplementary figures legend**

Supplementary Figure S.1.: Group-average functional and metabolic features. Dynamic [^18^F]FDG-PET data were used to estimate a) FDG micro/macro-parameters (*K*_1_, *k*_3_ and *K*_i_) at nodal level (86 ROIs) and c) Metabolic Connectivity (MC) matrices at network level (7 RSNs per hemisphere plus 12 SUBCORTICAL regions). From rs-fMRI data, both in F_1_ and F_2_ band, we derived c) Functional Connectivity (FC) matrices at network level (7 RSNs per hemisphere plus 12 SUBCORTICAL regions) and b) FC strength (*FC_STR_*) for each of the 86 ROIs. Brain nodes were grouped based on RSNs (left-hemisphere nodes (L) first, followed by right-hemisphere ones (R)). VIS = visual network; SOM = somatomotor network; DAN= Dorsal attention network; SALVENT = Salience/Ventral attention network; LIMBIC = Limbic network; CONT = Control network; DMN= Default mode network; SUBCORTICAL = thalamus, caudate, putamen, pallidum, cerebellum and hippocampus.

Supplementary Figure S.2.: Full matrices of PLSC saliences at network-level. The colours of the entries correspond to the magnitude and sign of the associated significant salience value (i.e., red for positive and grey for negative saliences’ values), with non-significant entries in white (according to the bootstrap procedure described in the Methods section).

Supplementary Figure S.3.: Fisher-z mean and 80^th^ percentile-thresholded FC in the F_2_ band. The figure displays the mean 80^th^ percentile-thresholded FC matrices for the group of *K*_1_-*FC_STR_* negative scores’ (on the left) and for the group of *K*_1_-*FC_STR_* positive scores’ (on the right)_,_ obtained by averaging the FC in the F_2_ band of the subjects belonging to each of the scores’ groups.

Supplementary Figure S.4.: Full-entries scores’ groups average FC and MC matrices. a) Mean FC (on the left) and MC (on the right) matrices for the group of subjects with positive FC-MC scores (a) and with negative FC-MC scores (b), obtained including only the significant FC and MC entries according to the corresponding PLSC salience map (non-significant values are shown in white).

Supplementary Figure S.5: Sensitivity analysis: effect of partial volume correction (PVC) on MC matrices (n = 42 subjects; n = 3,655 pairwise connections). (A) Group-average difference matrix (post − pre PVC; diverging red–blue colormap centred at zero), showing the spatial distribution of PVC-induced changes across all 86 ROIs. Warm colours indicate connections with increased MC after correction; cool colours indicate decreased MC. (B) Distribution of per-subject Spearman rank correlations between pre- and post-PVC MC vectors (red line: group mean r = 0.639; dashed line: r = 0.5 reference threshold); 4 out of 42 subjects showed r < 0.5; all p-values <0.0001). (C) Scatter of group-average pre- vs post-PVC MC values for all pairwise connections, coloured by symmetric relative change (hot colormap); solid line: mean subject-level regression fit (slope = 0.582, intercept = 0.246); dashed line: identity. The systematic deviation from the identity line reflects both a scale change and an additive offset introduced by PVC. (D) Distribution of mean symmetric relative change per connection (|post − pre| / (0.5 · |post + pre|)); white line: mean (25.3%); dashed line: median. (E) Symmetric matrix of mean relative change per connection; the concentration of high-change values along rows and columns corresponding to subcortical ROIs is clearly visible. (F) Top 15 ROIs ranked by mean relative change averaged across all their connections; colour intensity reflects the magnitude of change. Subcortical structures (Caudate, Pallidum, Hippocampus) consistently show the largest PVC-induced corrections, consistent with their known high susceptibility to partial volume effects due to small volume and grey matter heterogeneity. Cortical nodes appearing among the most affected ROIs (Cont_pCun, Default_PCC_RSC) reflect their disproportionate share of cortico-subcortical connections rather than intrinsic cortical PVE.

**References**

1.	Power JD, Barnes KA, Snyder AZ, Schlaggar BL, Petersen SE. Spurious but systematic correlations in functional connectivity MRI networks arise from subject motion. *NeuroImage*. 2012;59(3):2142-2154. doi:10.1016/j.neuroimage.2011.10.018

2.	Volpi T, Vallini G, Silvestri E, et al. A new framework for metabolic connectivity mapping using bolus [^18^ F]FDG PET and kinetic modeling. *J Cereb Blood Flow Metab*. 2023;43(11):1905-1918. doi:10.1177/0271678X231184365

3.	Bai P, Shen H, Huang X, Truong Y. A supervised singular value decomposition for independent component analysis of fMRI. *Statistica Sinica*. Published online 2008:1233-1252.

4.	Takane Y. Relationships among various kinds of eigenvalue and singular value decompositions. In: *New Developments in Psychometrics: Proceedings of the International Meeting of the Psychometric Society IMPS2001. Osaka, Japan, July 15–19, 2001*. Springer; 2003:45-56.

5.	Zöller D, Kebets V. myPLS - Partial least squares analysis for medical image processing.

6.	Erlandsson K, Buvat I, Pretorius PH, Thomas BA, Hutton BF. A review of partial volume correction techniques for emission tomography and their applications in neurology, cardiology and oncology. *Phys Med Biol*. 2012;57(21):R119-159. doi:10.1088/0031-9155/57/21/R119

7.	Thomas BA, Cuplov V, Bousse A, et al. PETPVC: a toolbox for performing partial volume correction techniques in positron emission tomography. *Phys Med Biol*. 2016;61(22):7975-7993. doi:10.1088/0031-9155/61/22/7975

8.	Tsoumpas C, Turkheimer FE, Thielemans K. Study of direct and indirect parametric estimation methods of linear models in dynamic positron emission tomography. *Medical Physics*. 2008;35(4):1299-1309. doi:10.1118/1.2885369

9.	Soret M, Bacharach SL, Buvat I. Partial-Volume Effect in PET Tumor Imaging. *Journal of Nuclear Medicine*. 2007;48(6):932-945. doi:10.2967/jnumed.106.035774

10.	Rousset OG, Ma Y, Evans AC. Correction for Partial Volume Effects in PET: Principle and Validation. *Journal of Nuclear Medicine*. 1998;39(5):904-911.

11.	Frouin V, Comtat C, Reilhac A, Grégoire MC. Correction of Partial-Volume Effect for PET Striatal Imaging: Fast Implementation and Study of Robustness. *Journal of Nuclear Medicine*. 2002;43(12):1715-1726.

12.	Greve DN, Salat DH, Bowen SL, et al. Different Partial Volume Correction Methods Lead to Different Conclusions: an 18F-FDG PET Study of Aging. *Neuroimage*. 2016;132:334-343. doi:10.1016/j.neuroimage.2016.02.042

13.	Quarantelli M, Berkouk K, Prinster A, et al. Integrated Software for the Analysis of Brain PET/SPECT Studies with Partial-Volume-Effect Correction. *Journal of Nuclear Medicine*. 2004;45(2):192-201.

14.	Labbé C, Froment JC, Kennedy A, Ashburner J, Cinotti L. Positron emission tomography metabolic data corrected for cortical atrophy using magnetic resonance imaging. *Alzheimer Dis Assoc Disord*. 1996;10(3):141-170. doi:10.1097/00002093-199601030-00005

15.	Baete K, Nuyts J, Van Paesschen W, Suetens P, Dupont P. Anatomical-based FDG-PET reconstruction for the detection of hypo-metabolic regions in epilepsy. *IEEE Transactions on Medical Imaging*. 2004;23(4):510-519. doi:10.1109/TMI.2004.825623

16.	Buckner RL, Andrews‐Hanna JR, Schacter DL. The brain’s default network: anatomy, function, and relevance to disease. *Annals of the new York Academy of Sciences*. 2008;1124(1):1-38.

17.	Hagmann P, Cammoun L, Gigandet X, et al. Mapping the Structural Core of Human Cerebral Cortex. *PLOS Biology*. 2008;6(7):e159. doi:10.1371/journal.pbio.0060159
